# CK2 Phosphorylation of Human Papillomavirus 16 E2 on Serine 23 Promotes Interaction with TopBP1 and Is Critical for E2 Interaction with Mitotic Chromatin and the Viral Life Cycle

**DOI:** 10.1128/mBio.01163-21

**Published:** 2021-09-21

**Authors:** Apurva T. Prabhakar, Claire D. James, Dipon Das, Raymonde Otoa, Matthew Day, John Burgner, Christian T. Fontan, Xu Wang, Sarah H. Glass, Andreas Wieland, Mary M. Donaldson, Molly L. Bristol, Renfeng Li, Anthony W. Oliver, Laurence H. Pearl, Brian O. Smith, Iain M. Morgan

**Affiliations:** a Virginia Commonwealth University (VCU), Philips Institute for Oral Health Research, School of Dentistry, Richmond, Virginia, USA; b Cancer Research UK DNA Repair Enzymes Group, Genome Damage and Stability Centre, School of Life Sciences, University of Sussex, Brighton, United Kingdom; c VCU School of Dentistry, Department of Oral Diagnostic Sciences, Richmond, Virginia, USA; d Emory Vaccine Center, Emory University School of Medicine, Atlanta, Georgia, USA; e Department of Microbiology & Immunology, Emory University School of Medicine, Atlanta, Georgia, USA; f School of Veterinary Medicine, University of Glasgow, Bearsden, United Kingdom; g VCU Massey Cancer Center, Richmond, Virginia, USA; h Institute of Molecular, Cell & Systems Biology, University of Glasgow, Glasgow, United Kingdom; Northwestern University

**Keywords:** human papillomavirus, E2, TopBP1, BRD4, cervical cancer, head and neck cancer, life cycle, CK2, phosphorylation, assay, papillomavirus

## Abstract

During the human papillomavirus 16 (HPV16) life cycle, the E2 protein interacts with host factors to regulate viral transcription, replication, and genome segregation/retention. Our understanding of host partner proteins and their roles in E2 functions remains incomplete. Here we demonstrate that CK2 phosphorylation of E2 on serine 23 promotes interaction with TopBP1 *in vitro* and *in vivo* and that E2 is phosphorylated on this residue during the HPV16 life cycle. We investigated the consequences of mutating serine 23 on E2 functions. E2-S23A (E2 with serine 23 mutated to alanine) activates and represses transcription identically to E2-WT (wild-type E2), and E2-S23A is as efficient as E2-WT in transient replication assays. However, E2-S23A has compromised interaction with mitotic chromatin compared with E2-WT. In E2-WT cells, both E2 and TopBP1 levels increase during mitosis compared with vector control cells. In E2-S23A cells, neither E2 nor TopBP1 levels increase during mitosis. Introduction of the S23A mutation into the HPV16 genome resulted in delayed immortalization of human foreskin keratinocytes (HFK) and higher episomal viral genome copy number in resulting established HFK. Remarkably, S23A cells had a disrupted viral life cycle in organotypic raft cultures, with a loss of E2 expression and a failure of viral replication. Overall, our results demonstrate that CK2 phosphorylation of E2 on serine 23 promotes interaction with TopBP1 and that this interaction is critical for the viral life cycle.

## INTRODUCTION

Human papillomavirus (HPV) infection leads to around 5% of all human cancers, with HPV16 infection being responsible for 50% of cervical cancers and 80 to 90% of HPV-positive oropharyngeal cancers (1). The latter disease has increased dramatically in the last generation and represents an ongoing public health crisis with no specific antiviral therapeutics available for combating the disease (2–6). Identification of such therapeutics is a priority, and our lab focuses on enhancing the molecular understanding of the HPV16 life cycle in order to identify potential antiviral targets.

HPVs infect basal epithelial cells, and following cell division, the viral DNA locates to the cell nucleus (7). The viral genome then replicates to 20 to 50 copies per cell, and the infected cell begins to proliferate, promoted by the expression of E6 and E7 that target p53 and pRb (among other proteins), respectively (8, 9). During proliferation, the viral genome copy number is maintained at around 20 to 50 copies per cell, and in the upper layers of the epithelium, there is a replication amplification stage where the viral genome copy number increases. The viral structural proteins L1 and L2 are then expressed and encapsulate the viral DNA to form viral particles that egress from the upper layers of the epithelium (10–12).

Throughout the viral life cycle, there are two viral proteins that mediate replication of the viral genome, E1 and E2 (13–15). E2 is a DNA binding factor whose carboxyl-terminal domain forms homodimers that bind to three 12-bp palindromic DNA sequences surrounding the viral origin of replication in the long control region (LCR), adjacent to the transcriptional start site (16, 17). There is a fourth E2 target site in the LCR further upstream from the viral origin of replication. Following binding to its target sequences, E2 recruits the viral helicase E1 to the origin of replication via a protein-protein interaction (14–16). At the A/T-rich origin, E1 forms a dihexameric helicase complex that interacts with host DNA polymerases to initiate viral DNA replication (18–21). Furthermore, E2 has additional roles during the viral life cycle. E2 can regulate transcription from the viral genome and can either activate or repress transcription depending upon the E2 concentration (22). E2 also regulates transcription from the host genome, and this regulation is directly relevant to the viral life cycle (23, 24). The fourth function for E2 during the viral life cycle is to mediate viral genome segregation (25). During cell division, the 8-kbp episomal viral genome could be excluded from the nuclei of resulting daughter cells. To combat this, the virus has an active mechanism that retains viral genomes in daughter nuclei via hitchhiking onto the host chromatin during mitosis (25). E2 mediates this function by binding to the viral DNA via its carboxyl-terminal DNA binding domain and simultaneously binding to host chromatin via the E2 amino-terminal domain (25). For bovine papillomavirus 1 (BPV1) E2, the host receptor mediating interaction with mitotic chromatin is BRD4 (26–29). For high-risk HPV (HR-HPV) (those that cause cancer, including HPV16), the E2 proteins do not colocalize with BRD4 on mitotic chromatin, indicating that BRD4 may not be the mitotic receptor for these E2 proteins (16, 30, 31). We identified TopBP1 as a functional interacting partner for HPV16 E2 (32–36). TopBP1 regulates the interaction of E2 with host chromatin in interphase cells and colocalizes with TopBP1 on mitotic chromatin, indicating that TopBP1 is a candidate protein for mediating E2 interaction with mitotic chromatin (37).

TopBP1 is a multifunctional protein involved in several aspects of nucleic acid metabolism (38). It is part of the replication complex in mammalian cells, interacting with Treslin to promote the initiation of replication (39–43). TopBP1 contains nine BRCT (BRCA1 carboxyl-terminal) domains that act as hydrophobic pockets mediating interaction with cellular proteins, including proteins that are phosphorylated following cell signaling events and are involved in replication initiation and the DNA damage response (DDR) (44–65). TopBP1 is required for the activation of the ATR (ataxia-telangiectasia and Rad3-related) kinase via interaction with ATRIP (ATR-interacting protein), and TopBP1 is also a substrate for ATM (ataxia-telangiectasia mutated). Both ATM and ATR are activated during the viral life cycle in order to promote viral genome replication; therefore, TopBP1 is an essential mediator of the HPV16 life cycle (66–70). TopBP1 also has several roles during mitosis as it prevents transmission of DNA damage (including DNA double-strand breaks and catenated DNA) to G_1_ daughter cells (45, 47, 65, 71–74).

Our previous work identified a mutant of E2 that had a compromised interaction with TopBP1, asparagine 89 and glutamic acid 90 of E2 were mutated to tyrosine and valine, respectively (36). The change in nature from polar and charged to bulkier and more hydrophobic at the substituted residues disrupted the interaction between HPV16 E2 and TopBP1 (from now on, E2 will mean HPV16 unless stated otherwise). To gain a more mechanistic understanding of the E2-TopBP1 interaction and how it is regulated, we tested potential phosphorylation sites on E2 that mediate TopBP1 interaction, as TopBP1 binds a number of phosphorylated proteins via its BRCT domains (38). Here we demonstrate that CK2 phosphorylation of E2 serine 23 promotes the interaction between E2 and TopBP1 *in vitro* and *in vivo*. E2-S23A (an alanine substitution at position serine 23 which is defective in TopBP1 interaction) and E2-WT (wild-type E2) have similar transcription and replication functions in our transient assays. E2 recruits TopBP1 onto mitotic chromatin and results in increased expression of both proteins during this period of the cell cycle, the E2 S23A mutant fails to increase either TopBP1 or E2 protein levels during mitosis. Introduction of the E2-S23A mutation into the HPV16 genome results in a delay in human foreskin keratinocyte (HFK) immortalization compared with the wild-type genome. Organotypic raft cultures demonstrate disruption of the HPV16 life cycle due to mutation of serine 23. Our results demonstrate a critical role for CK2 phosphorylation of E2-S23 that is important for TopBP1 interaction and mitotic chromatin interaction. Together, our studies suggest that the E2-TopBP1 interaction is critical at multiple points of the HPV16 life cycle.

## RESULTS

### E2 serine 23 is critical for TopBP1 interaction *in vivo*.

**FIG 1:**
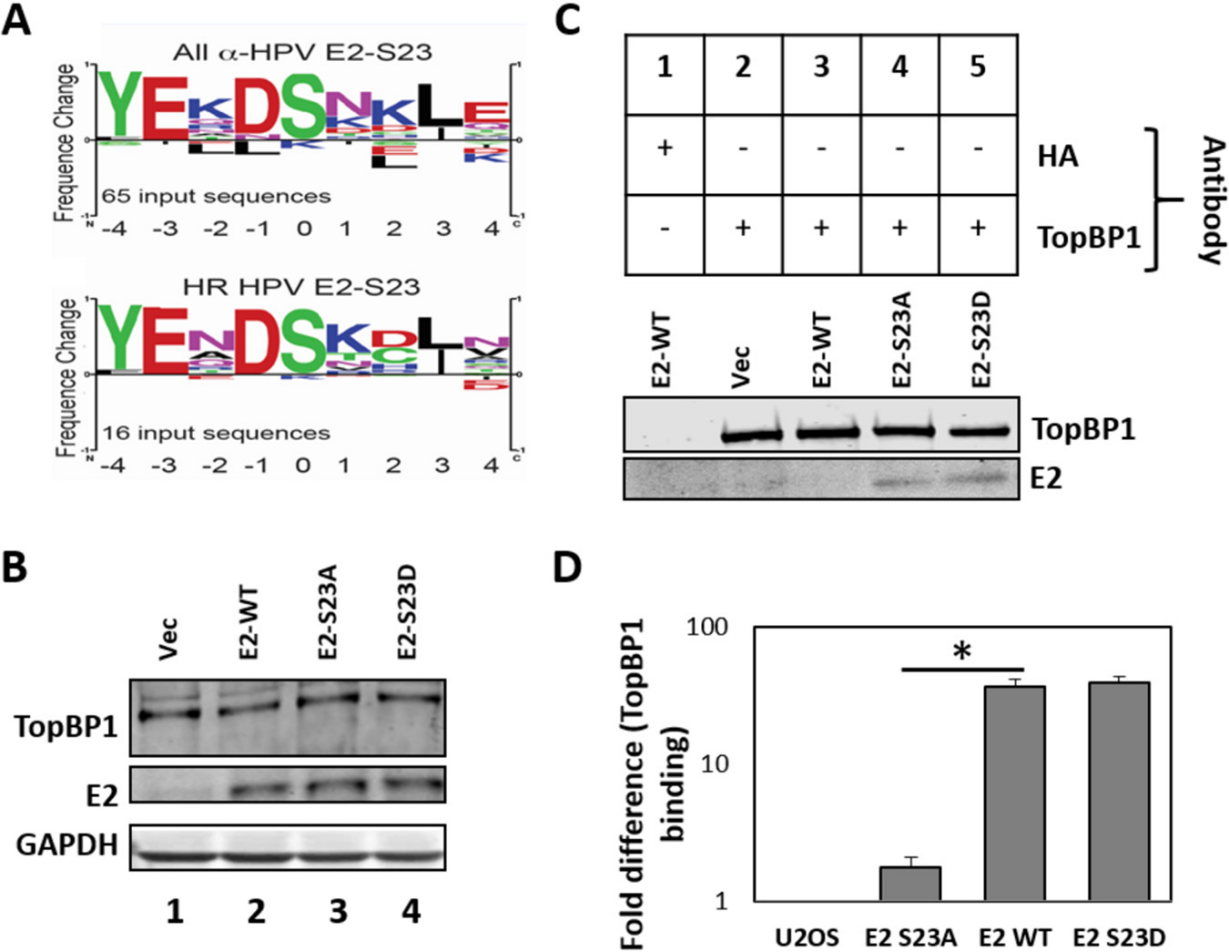
(A) Motif analysis of the E2 serine 23 residue region in all α-HPV (top) and high-risk (HR) (cancer causing) HPV (bottom). (B) Western blots of U2OS cells expressing the indicated E2 proteins. Vec, vector. (C) Immunoprecipitation with HA (control) and TopBP1 antibody followed by Western blotting for TopBP1 and E2. (D) Quantitation of repeat TopBP1 co-IPs. E2-specific antibody TVG261 (ab17185) was used for Western blotting in panels B and C. The asterisk indicates a significant decrease in E2-S23A interaction with TopBP1 compared with E2-WT (*P* value < 0.05).

**FIG 2:**
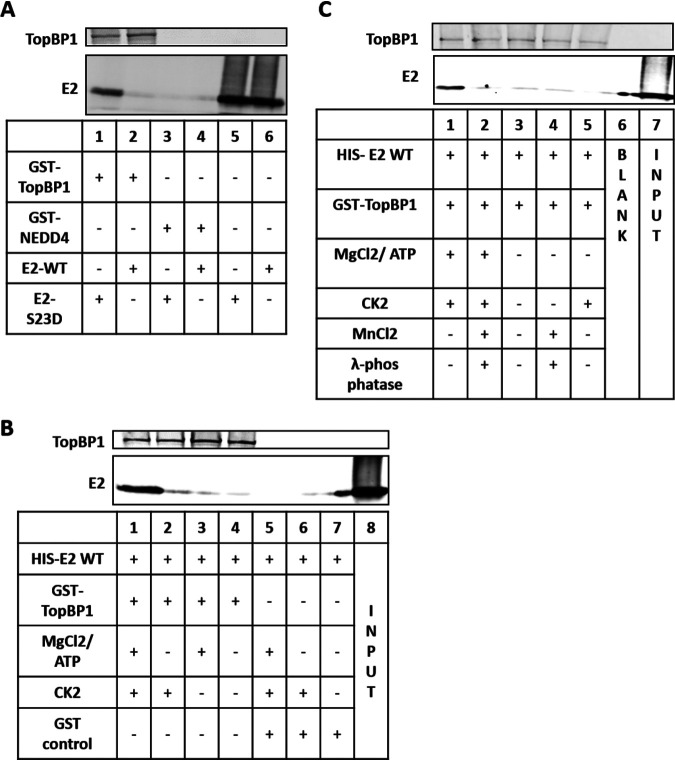
(A) GST-TopBP1 or GST-NEDD4 (0.65 pmol) was incubated with 11 pmol E2 and incubated at 4°C for 1 h with rotation. GST pulldowns followed by Western blotting for TopBP1 (top blot) and E2 (bottom blot) were then carried out. (B) The GST pulldown was repeated as in panel A following 1 h at 30°C with CK2 and controls. (C) Lambda phosphatase was added to the CK2 reaction mixture, and GST pulldown assays were carried out as in panel A. E2-specific antibody TVG261 (ab17185) was used for Western blotting in all three experiments (A to C). Figure S1A to S1C in the supplemental material summarizes quantitation of repeat experiments. In the input lanes, only E2 was added. The TopBP1 pulldown demonstrates equivalent levels of TopBP1 in each reaction mixture containing TopBP1.

Because TopBP1 binds to phosphorylated proteins, we investigated the ability of potential phosphorylation sites on E2 to mediate the interaction with TopBP1. E2 protein sequence analysis showed that serine 23 is highly conserved in alpha-type HPV (that incorporate high-risk HPV [HR-HPV]) (Fig. 1A). Also, on the crystal structure model for HPV16 E2, serine 23 juxtaposes with amino acids 89 and 90, mutation of which disrupts E2-TopBP1 interaction (36, 75). To investigate the interaction between E2 and TopBP1 via serine 23, U2OS cells stably expressing E2-WT (wild-type), E2-S23A (serine mutated to alanine), and E2-S23D (serine mutated to aspartic acid) were generated, along with pcDNA empty vector plasmid control (Fig. 1B). Cell extracts from Fig. 1B were immunoprecipitated by a TopBP1 antibody, and TopBP1 and E2 were detected using Western blotting (Fig. 1C); this experiment was repeated on three independent extracts and quantitated (Fig. 1D). While E2-WT and E2-S23D coprecipitate with TopBP1 (Fig. 2C, lanes 4 and 5), E2-S23A is significantly compromised in this interaction (Fig. 1C, lane 3, and Fig. 1D).

10.1128/mBio.01163-21.1 FIG S1 (A) This is a quantitation of repeat experiments shown in Fig. 2A, and the binding of E2 to TopBP1 is expressed relative to input levels equaling 1. (B) This is a quantitation of repeat experiments shown in Fig. 2B. The binding of E2 to TopBP1 is expressed relative to the E2 input protein equaling 1. (C) This is a quantitation of repeat experiments sown in Fig. 2C. The binding of E2 to TopBP1 is repressed relative to the E2 input protein equaling 1. An asterisk indicates a significant difference between the two samples under the bracket (*P* value < 0.05). Significance was determined using a Student’s *t* test, and standard errors were calculated from three independent experiments. Download FIG S1, TIF file, 0.10 MB. Copyright © 2021 Prabhakar et al. 2021 Prabhakar et al. https://creativecommons.org/licenses/by/4.0/ This content is distributed under the terms of the Creative Commons Attribution 4.0 International license.

### CK2 phosphorylation of E2 promotes interaction with TopBP1 *in vitro* and *in vivo*.

The negative charges at positions −1 and −3 in the E2 consensus sequence around serine 23 (Fig. 1A) indicate a potential CK2 target site at serine 23 (76). CK2 is also active during mitosis and could therefore be involved in mediating the plasmid retention function of E2 (77). CK2 interacts with E2, and TopBP1 interacts with CK2 phosphorylated proteins; therefore, we investigated whether CK2 phosphorylates E2 serine 23 (59, 78–80). We prepared recombinant GST-TopBP1 (full length), His-E2-WT, and His-E2-S23D (amino acids 1 to 200 for both E2 proteins) from bacteria. These proteins were incubated together, and glutathione *S*-transferase (GST) pulldown experiments were carried out followed by Western blotting (Fig. 2A). Lanes 5 and 6 in Fig. 2A demonstrate equivalent levels of E2-S23D and E2-WT input in the GST interactions. Lane 1 demonstrates an interaction between E2-S23D and GST-TopBP1, while lane 2 demonstrates that E2-WT does not interact with TopBP1. Neither protein interacts with the GST-NEDD4 control protein. This experiment was repeated, and the results were quantitated (see Fig. S1A in the supplemental material). To determine whether CK2 phosphorylation of E2-WT can promote interaction with TopBP1, we incubated the recombinant proteins with CK2 enzyme prior to the GST pulldown experiments (Fig. 2B). Lane 1 in Fig. 2B demonstrates that the presence of an enzymatically active CK2 promotes interaction between E2-WT and TopBP1. Omission of CK2 cofactors (MgCl_2_/ATP) (lane 2) or CK2 enzyme (lane 3) abolished the interaction. CK2 did not promote interaction with GST-NEDD4 (present in lanes 5 to 7). This experiment was repeated, and the results were quantitated (Fig. S1B). To confirm that it is CK2 phosphorylation promoting the interaction between E2-WT and TopBP1, we repeated the experiment in Fig. 2B in the presence of lambda phosphatase which eliminates the interaction between E2 and TopBP1 (Fig. 2C, lane 2). This experiment was repeated, and the results were quantitated (Fig. S1C). Unfortunately, we were unable to produce recombinant E2 S23A protein, despite repeated tries and confirmation that appropriate RNA was being produced in the bacteria following induction.

**FIG 3:**
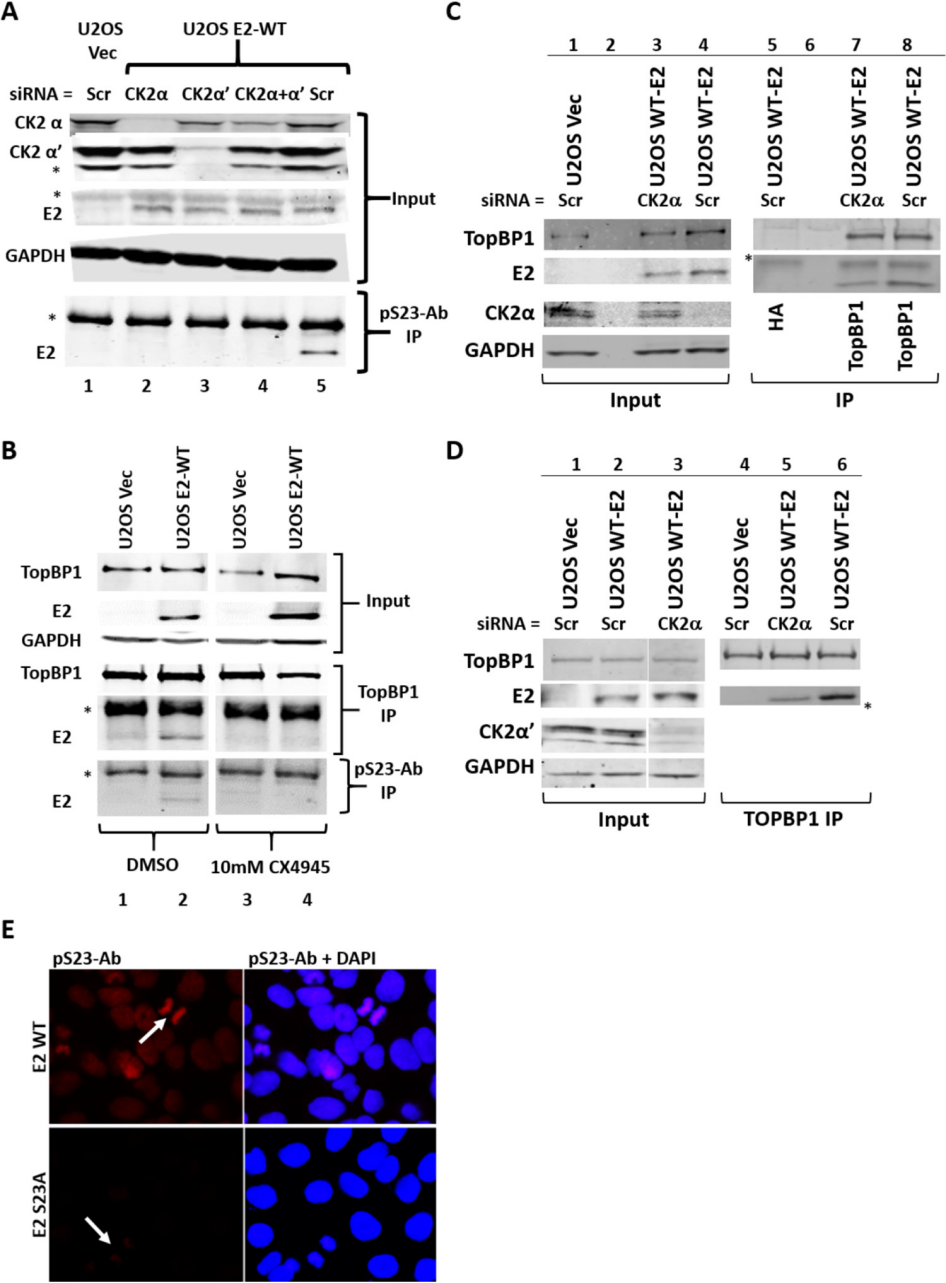
(A) siRNA knockdown of CK2α and/or CK2α′. Scr control siRNA was used in lanes 1 and 5. The top panels demonstrate the input proteins that were used in the immunoprecipitation (IP) with pS23-Ab (an antibody raised against an E2 peptide containing a phosphorylated serine 23). Please note the CK2α blot is independent of the other inputs but is run with the same protein extracts. The IP was blotted for E2 which is clearly detected in the Scr control (lane 5), but not in the CK2 knockdown cells (lanes 2 to 4). (B) The indicated cells were treated with DMSO (lanes 1 and 2) or the CK2 inhibitor CX4945 (lanes 3 and 4), and Western blotting details the levels of TopBP1 and E2 in the treated cells (top blots). Immunoprecipitation (IP) with TopBP1 demonstrates a pulldown of TopBP1 and E2 (lane 2) that is abrogated by CX4945 (lane 4) (middle blots). IP with pS23-Ab pulled down E2 in control cells (lane 2) that was abolished by CX4945 (lane 4). (C) CK2a siRNA knockdown (lane 4) disrupted the E2-TopBP1 interaction as there was a reduced co-IP of E2 in the absence of CK2α (lane 7). (D) CK2α′ knockdown (lane 3) disrupted the E2-TopBP1 interaction as there was a reduced co-IP of E2 in the absence of CK2α′ (lane 5). Please note that lanes 1 to 3 are from the same gel and the same exposure with a lane removed. (E) Staining of U2OS E2-WT (top panels) and U2OS E2-S23A (bottom panels) with pS23-Ab. Left-hand panels are antibody only, right-hand panels are antibody plus DAPI. There was no signal generated with secondary antibody only, and no signal detected in U2OS-Vec control when the primary antibody was included. HPV16 E2 B9 monoclonal antibody was used for Western blotting in panels A and B. E2-specific antibody TVG261 (ab17185) was used for Western blotting in panels C and D. Figure S2A and S2B summarizes quantitation for repeat experiments of panels C and D, respectively. An asterisk indicates an antibody band.

To investigate whether E2 S23 is phosphorylated *in vivo* by CK2, we generated an antibody specific for phosphorylated serine 23 (pS23-Ab) using a phospho-peptide incorporating the region around S23 with the serine phosphorylated (CKILTHYENDS^P^TDLR). To investigate whether CK2 was responsible for phosphorylating serine 23 *in vivo*, we knocked down CK2 components using small interfering RNA (siRNA). Figure 3A demonstrates that expression of CK2α and CK2α′ was downregulated using siRNA (lanes 2 and 3, respectively); both were also targeted (lane 4). Nonspecific scrambled control siRNA (Scr) was used as a control for siRNA treatment in U2OS-Vec and U2OS-E2-WT (lanes 1 and 5, respectively). When both siRNAs were combined, there was a partial knockdown of both proteins. There was visible toxicity in the double knockdown cells, and the partial knockdown is likely due to a survival advantage of cells exhibiting a lower degree of both CK2 component knockdown. Following immunoprecipitation with pS23-Ab, there is coimmunoprecipitation (co-IP) of E2 in the Scr-treated U2OS-E2-WT cells (lane 5, lower blot). Under all conditions when CK2 components are targeted by specific siRNAs, there is an abrogation of detectable E2 co-IP with the pS23-Ab (lanes 2 to 4). To confirm the role of CK2 in phosphorylating E2 S23 to promote interaction with TopBP1, we used the CK2 inhibitor CX4945 (Fig. 3B). In the presence of CX4945 (lanes 3 and 4), the interaction between E2 and TopBP1 is disrupted, and pS23-Ab fails to co-IP E2. To confirm that knockdown of CK2 components disrupts the E2-TopBP1 interaction, we carried out TopBP1 co-IPs following CK2α or CK2α′ siRNA knockdown. Figure 3C (lanes 2 and 6 are blank) demonstrates that knockdown of CK2α reduces the interaction between E2 and TopBP1. There is knockdown of CK2α expression with the targeting siRNA, while Scr (scrambled) control has robust CK2α expression (compare lane 4 with lanes 1 and 3). The hemagglutinin (HA) control antibody IP does not immunoprecipitate TopBP1 or E2 (lane 5), while TopBP1 antibody pulls down both proteins (lanes 7 and 8). There is a reduced co-IP of E2 with the TopBP1 antibody when CK2α is knocked down (compare lane 7 with lane 8). This experiment was repeated and quantitated, and there is a statistically significant reduction in the interaction between TopBP1 and E2 following knockdown of CK2α expression (Fig. S2A). Figure 3D demonstrates that knockdown of CK2α′ expression also reduces the E2-TopBP1 interaction. Lane 3 (taken from the same gel as lanes 1 and 2 with lanes removed) demonstrates a reduction of CK2α′ with targeting siRNA compared with the control Scr siRNA. Immunoprecipitation with TopBP1 antibody results in a co-IP with E2 (lanes 5 and 6). As with CK2α, there is a reduction in the amount of E2 co-IP with TopBP1 when CK2α′ is knocked down (compare lane 5 with lane 6). These experiments were repeated and quantitated, and there is a statistically significant reduction in the interaction between TopBP1 and E2 following knockdown of CK2α′ expression (Fig. S2B). The E2-TopBP1 interaction is not completely eliminated because CK2α and CK2α′ likely compensate for the absence of the other.

10.1128/mBio.01163-21.2 FIG S2 (A) This is a quantitation of repeat experiments from Fig. 3C. The co-IPs of E2 with TopBP1 in the presence of Scr and CK2α siRNA were quantitated relative to the input protein levels. The Scr co-IP levels were set at 1. (B) This is a quantitation of repeat experiments from Fig. 3C. The co-IPs of E2 with TopBP1 in the presence of Scr and CK2α′ siRNA were quantitated relative to the input protein levels. The Scr co-IP levels were set at 1. An asterisk indicates a significant difference between the two samples under the bracket (*P* value < 0.05). Significance was determined using a Student’s *t* test, and standard errors weres calculated from three independent experiments. Download FIG S2, TIF file, 0.07 MB. Copyright © 2021 Prabhakar et al. 2021 Prabhakar et al. https://creativecommons.org/licenses/by/4.0/ This content is distributed under the terms of the Creative Commons Attribution 4.0 International license.

To investigate whether E2 is phosphorylated on serine 23 during mitosis, we prepared mitotically enriched U2OS E2-WT and E2-S23A cells. Figure 3E demonstrates a strong signal in the E2-WT mitotic and interphase cells following pS23-Ab staining. With E2-S23A cells, staining with pS23-Ab generates no visible staining in interphase cells, and a very marginal signal in mitotic cells. Figure 5 clearly shows that the E2-S23A protein is detectable by immunofluorescence in these cells with a non-phospho-specific E2 antibody, and Fig. 1B demonstrates equivalent expression levels of E2-WT and E2-S23A in U2OS cells. Overall, Fig. 2 and 3 demonstrate that CK2 phosphorylates E2 on serine 23 to promote interaction with TopBP1.

### CK2 phosphorylates E2 serine 23 in human keratinocytes.

**FIG 4:**
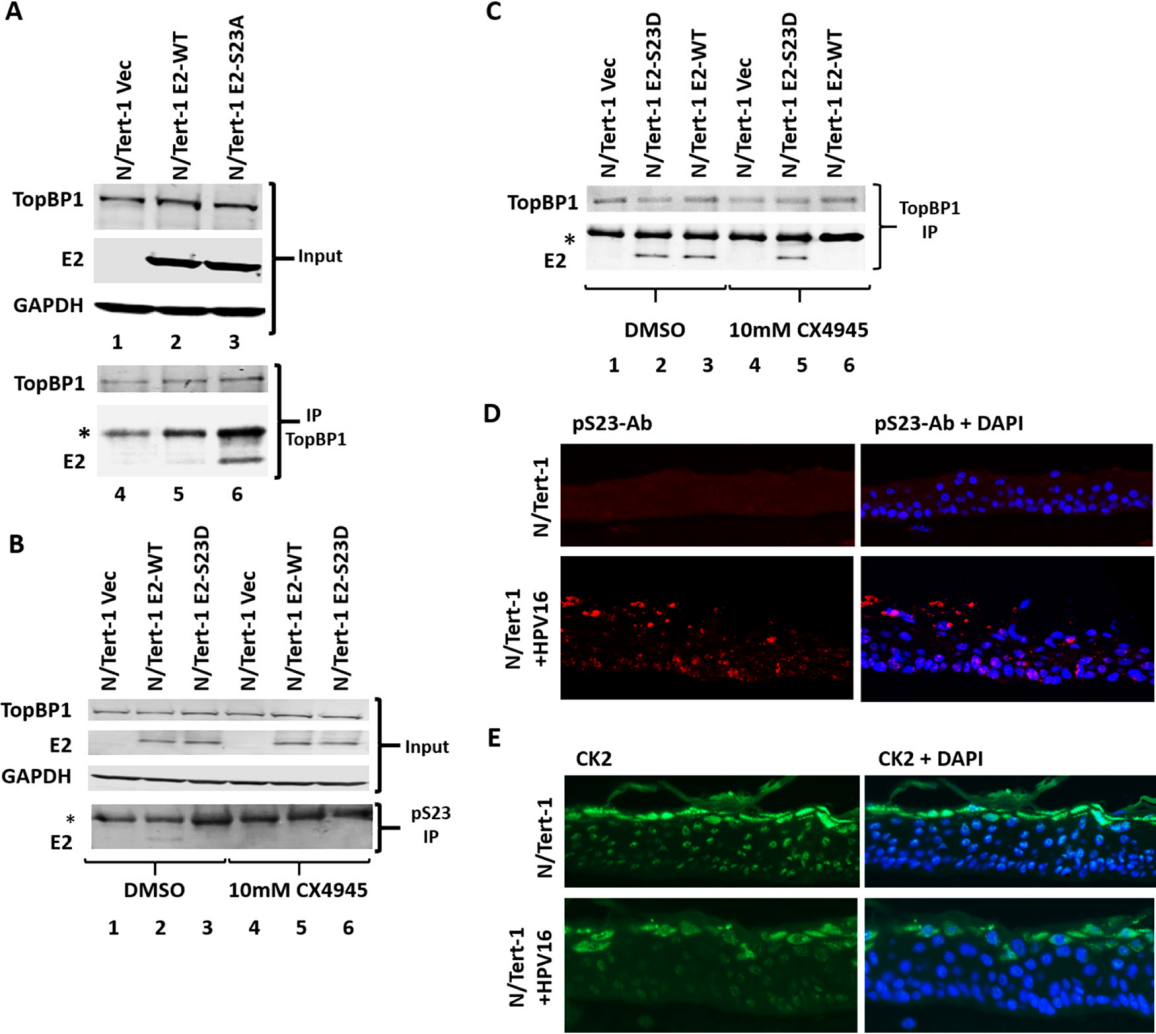
(A) Top blots with lanes 1 to 3 are Western blots of extracts from the indicated stable N/Tert-1 cell lines. Bottom blots with lanes 4 to 6 are Western blots of a TopBP1 immunoprecipitation (IP) of the indicated extracts. TopBP1 co-IPs E2-WT but not E2-S23A. (B) The extracts in the top blots (Input) were immunoprecipitated with pS23-Ab, and E2 is pulled down by this antibody (bottom blot, lane 2). The CK2 inhibitor CX4945 abrogates this pulldown (lane 5). (C) The extracts from panel B were immunoprecipitated with TopBP1, and both E2-WT and E2-S23D co-IP with TopBP1 (lanes 2 and 3). Treatment with the CX4945 abrogates the interaction between TopBP1 and E2-WT (lane 6), but not E2-S23D (lane 5). (D) Organotypic raft cultures of N/Tert-1 (top panels) and N/Tert-1+HPV16 (bottom panels) were stained with pS23-Ab. There is no specific staining in N/Tert-1 cells, but there is clear staining in N/Tert-1+HPV16. (Left panels) pS23-Ab only, (right panels) pS23-Ab plus DAPI staining. An asterisk indicates an antibody band. (E) Organotypic raft cultures of N/Tert-1 (top panels) and N/Tert-1+HPV16 (bottom panels) stained with CK2 antibody. HPV16 E2 B9 monoclonal antibody was used for Western blotting in panels A to C.

We extended our studies into human keratinocytes, the natural target cell type for HPV16 infection. We established N/Tert-1 (human foreskin keratinocytes immortalized by human telomerase reverse transcriptase [hTERT]) cells expressing E2-WT and E2-S23A, as described previously, along with pcDNA Vec control (24). Figure 4A demonstrates expression of E2-WT and E2-S23A in the N/Tert-1 cells (compare lanes 2 and 3, respectively, with lane 1). Immunoprecipitation brought down TopBP1 in all lines (lanes 4 to 6, top blot), but only E2-WT interacted strongly with TopBP1, demonstrating that the E2-S23A mutation also disrupted the E2-TopBP1 interaction in N/Tert-1 cells (compare lane 6 with lane 5). Figure 4B demonstrates that the pS23-Ab recognizes E2-WT in N/Tert-1 cells; IP with pS23-Ab pulls down E2 (lane 2). The addition of CX4945 (the CK2 inhibitor) to the N/Tert-1 cells abolished E2-WT pulldown with pS23-Ab (compare lane 5 with lane 2), demonstrating that CK2 is responsible for the phosphorylation of E2 serine 23 in N/Tert-1 cells. Abrogation of E2 serine phosphorylation by CX4945 disrupted the E2-TopBP1 interaction (Fig. 4C; the inputs from Fig. 4B were used). TopBP1 pulled down both E2-WT and E2-S23D (as it does in U2OS cells [Fig. 1]) (Fig. 4C, lanes 2 and 3). Treatment with CX4945 disrupted the interaction between TopBP1 and E2-WT but had no effect on the interaction with TopBP1 interaction with E2-S23D. Overall, these results demonstrate that the E2-TopBP1 interaction in N/Tert-1 cells is mediated by CK2 phosphorylation of serine 23 in N/Tert-1 cells.

To investigate whether E2 S23 is phosphorylated during the HPV16 life cycle, we stained N/Tert-1 and N/Tert-1+HPV16 organotypic raft cultures with the pS23-Ab. Our previous work demonstrates that N/Tert-1+HPV16 cells have episomal HPV16 genomes and support late stages of the HPV16 life cycle (24, 81). Figure 4D demonstrates that E2 is phosphorylated on serine 23 in the N/Tert-1+HPV16 cells (bottom panels). The N/Tert-1-Vec cells serve as an isogenic control line; there is no positive signal generated with the pS23-Ab in these cells (top panels). Staining in N/Tert-1+HPV16 is detected throughout the epithelial layer and is clearly nuclear in many of the cells. Please note that the staining outside of nuclei likely relates to nuclear breakdown in the upper layers of the differentiated epithelium, and also potentially reflect “smear” artifacts introduced during microsectioning. There is positive staining that is nuclear in many of the cells in N/Tert-1+HPV16. CK2α staining revealed nuclear expression of this protein in N/Tert-1 and N/Tert-1+HPV16, although there appeared to be less protein detected in the latter, indicating that HPV16 may control the expression of this protein during the viral life cycle (Fig. 4E).

### The E2 S23A mutation disrupts E2 interaction with mitotic chromatin.

E2 has three clear roles in the viral life cycle: regulation of transcription from the viral and host genomes, replication of the viral genome in association with E1, and segregation of the viral genome into daughter cells where it acts as a bridge between the viral and host genomes during mitosis. The latter function guarantees that the viral genomes are retained in daughter nuclei following mitosis. We measured the transcriptional activation potential of E2-WT and E2-S23A using our ptk6E2-luc system, a plasmid with six E2 sites located upstream from the herpes simplex virus 1 (HSV-1) thymidine kinase (tk) promoter driving expression of luciferase (luc) (82, 83). Both E2-WT and E2-S23A were able to activate transcription from this reporter with no significant difference in activity (Fig. S3A). E2 can repress transcription from the HPV16 long control region (LCR), and E2-WT and E2-S23A were equally able to repress transcription from our pHPV16-LCR-luc reporter (84) (Fig. S3B). Using our transient E1-E2 DNA replication assay, we demonstrated that both E2-WT and E2-S23A were able to activate replication with no significant difference between them (85) (Fig. S3C). In support of this, E2-S23A was able to interact with E1 similarly to E2-WT (Fig. S4).

10.1128/mBio.01163-21.3 FIG S3 (A) U2OS cells were transfected with the indicated plasmids (no E2 had a pcDNA control to maintain identical DNA concentrations in all samples) and luciferase assays carried out on cell extracts from the transfected cells. The luciferase activity was standardized to protein levels in the cell extract. The figure represents a summary of three independent experiments carried out in duplicate. There was no significant difference between the transcriptional activation properties of E2-WT or E2-S23A. (B) U2OS cells were transfected with the indicated plasmids (no E2 had a pcDNA control to maintain identical DNA concentrations in all samples) and luciferase assays carried out on cell extracts from the transfected cells. The luciferase activity was standardized to protein levels in the cell extract. The figure represents a summary of three independent experiments carried out in duplicate. There was no significant difference between the transcriptional repression properties of E2-WT or E2-S23A. For a more detailed description of how these assays were carried out, see reference 84. (C) C33a cells were transfected with the indicated plasmids, low molecular weight DNA was harvested after 48 h, and replication levels were determined as described previously (85). There is no replication with E1 alone; E2 and E1 are required for replication in this assay. There was no statistically significant difference between the replication levels of E2-WT or E2-S23A. Download FIG S3, TIF file, 0.1 MB. Copyright © 2021 Prabhakar et al. 2021 Prabhakar et al. https://creativecommons.org/licenses/by/4.0/ This content is distributed under the terms of the Creative Commons Attribution 4.0 International license.

10.1128/mBio.01163-21.4 FIG S4 E2 S23A binds to E1. C33a cells were transfected with 1 μg of the indicated expression plasmids, and protein extracts were prepared 48 h later. Inputs were determined by Western blotting (top panel), while the interaction between E2 and E1 was determined using HA immunoprecipitation (bottom panel; the E1 is HA tagged). The S23A mutant interacts efficiently with E1 (lane 8). Download FIG S4, TIF file, 0.1 MB. Copyright © 2021 Prabhakar et al. 2021 Prabhakar et al. https://creativecommons.org/licenses/by/4.0/ This content is distributed under the terms of the Creative Commons Attribution 4.0 International license.

**FIG 5:**
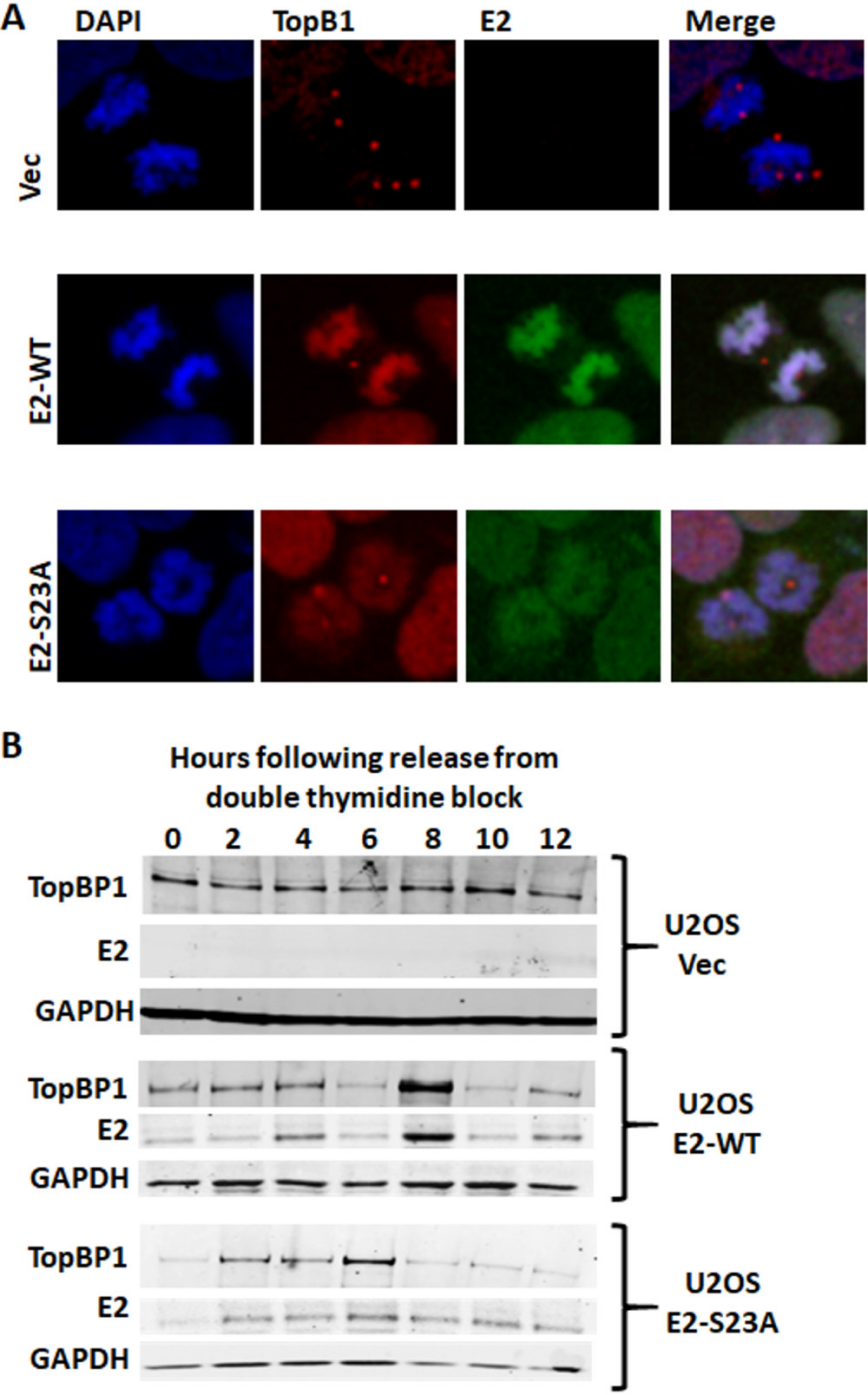
(A) U2OS-Vec (top panels), E2-WT (middle panels), and E2-S23A were stained with TopBP1 or E2 as indicated. In the right-hand panels, a merge of the two antibodies with DAPI staining is shown. TopBP1 does not specifically associate with mitotic chromatin in the Vec cells, but it does in the E2-WT and E2-S23A cells. The staining seemed more intense for E2 and TopBP1 with E2-WT than with E2-S23A. These are representative images from experiments where similar phenotypes are repeatedly observed in all three lines. (B) The indicated U2OS lines were double thymidine blocked to coordinate them in G_1_. They were then released at the indicated time points, and protein extracts were harvested and Western blotting was carried out. At the 8-h time point, there is an enrichment for cells in mitosis in all cell lines (Fig. S5A to S5C). These results were repeated and quantitated, and there was a significant increase of E2 and TopBP1 at the 8-h time point only in U2OS E2-WT cells (Fig. S5D). E2-specific antibody TVG261 (ab17185) was used for immunofluorescence staining in panel A and Western blotting in panel B.

We next investigated the role of TopBP1 in E2 interaction with mitotic chromatin. U2OS cells have excellent nuclear architecture and also support HPV replication and the maintenance of episomal genomes (86). We synchronized the U2OS cells to enrich for mitotic cells, and Fig. 5A shows representative images from cells stained with 4′,6′-diamidino-2-phenylindole (DAPI), E2, and TopBP1. In the absence of E2, TopBP1 does not coat mitotic chromatin, instead showing punctate staining associated with the mitotic DNA, as observed previously (45) (Fig. 5A, top panels). E2-WT and TopBP1 (middle panels) colocalized on mitotic chromatin, demonstrating that the presence of E2 promotes TopBP1 interaction with the mitotic chromatin. The mutant E2-S23A localized to mitotic chromatin along with TopBP1, but the observed staining was less intense. This pattern of staining was similar in all mitotic cells from the three cell lines. The less intense staining of mitotic chromatin with E2-S23A, compared with E2-WT, suggests that the interaction between E2 and TopBP1 may elevate E2 protein levels during mitosis. To investigate this, U2OS cells were synchronized by double thymidine blocking, and protein was harvested from the cells every 2 h up until 12 h following release from blocking. Western blotting was then performed on protein extracts from the cell (Fig. 5B). Flow cytometry of the cell lines demonstrating their cell cycle status at different time points are shown in Fig. S5A to C. This experiment was repeated and the results for E2 and TopBP1 quantitated (Fig. S5D). Strikingly, there was a significant increase in E2 and TopBP1 levels 8 h following release in the E2-WT cells, but no increase of either E2 or TopBP1 in E2-S23A or Vec control cells. The 8-h time point correlates strongly with mitosis (Fig. S5). Overall, the results demonstrate that a major role for the E2-TopBP1 interaction is to promote increased levels of both proteins during mitosis. It is also clear that there are other factors that can regulate the interaction of E2 with chromatin, as E2-S23A still interacts with mitotic chromatin, and TopBP1 is also recruited to mitotic chromatin with E2-S23A. One potential host protein mediating this function is BRD4, and we demonstrate that BRD4 is able to interact with E2-S23A and that TopBP1 and BRD4 exist in the same cellular complex (Fig. S6).

10.1128/mBio.01163-21.5 FIG S5 (A, B, and C) Flow cytometry data for U2OS-Vec, U2OS E2-WT, and U2OS E2-S23A. The cells were double thymidine blocked (DTB) as described in Materials and Methods in the main article. Following release from the DTB, cells were harvested for flow cytometry analysis. Propidium iodide staining and flow cytometry analysis with a FACSAria fusion SORP high-speed cell sorter (Becton Dickinson), using FlowJo software, were used for the cell cycle phase analysis. (D) This is a quantitation of repeat experiments shown in Fig. 5B. The top panels show the levels of E2 relative to GAPDH at various times following release from double thymidine block, while the bottom panels show the levels of TopBP1 relative to GAPDH following release. An asterisk indicates a significant difference in protein levels between the 0- and 8-h time points. Significance was determined using a Student’s *t* test, and standard errors were calculated from three independent experiments. Download FIG S5, TIF file, 0.4 MB. Copyright © 2021 Prabhakar et al. 2021 Prabhakar et al. https://creativecommons.org/licenses/by/4.0/ This content is distributed under the terms of the Creative Commons Attribution 4.0 International license.

10.1128/mBio.01163-21.6 FIG S6 Protein extracts were prepared from U2OS cells containing vector control or E2-WT or E2-S23A and Western blotted for BRD4, TopBP1, E2, or GAPDH (A). These extracts were then subjected to immunoprecipitation with a BRD4 antibody, and the resulting IPs were blotted for BRD4, TopBP1, or E2 (B). The results demonstrate that BRD4 and TopBP1 form a complex irrespective of E2 expression and that both E2-WT and E2-S23A can interact with BRD4. Download FIG S6, TIF file, 0.3 MB. Copyright © 2021 Prabhakar et al. 2021 Prabhakar et al. https://creativecommons.org/licenses/by/4.0/ This content is distributed under the terms of the Creative Commons Attribution 4.0 International license.

### E2 serine 23 and the HPV16 life cycle.

**FIG 6:**
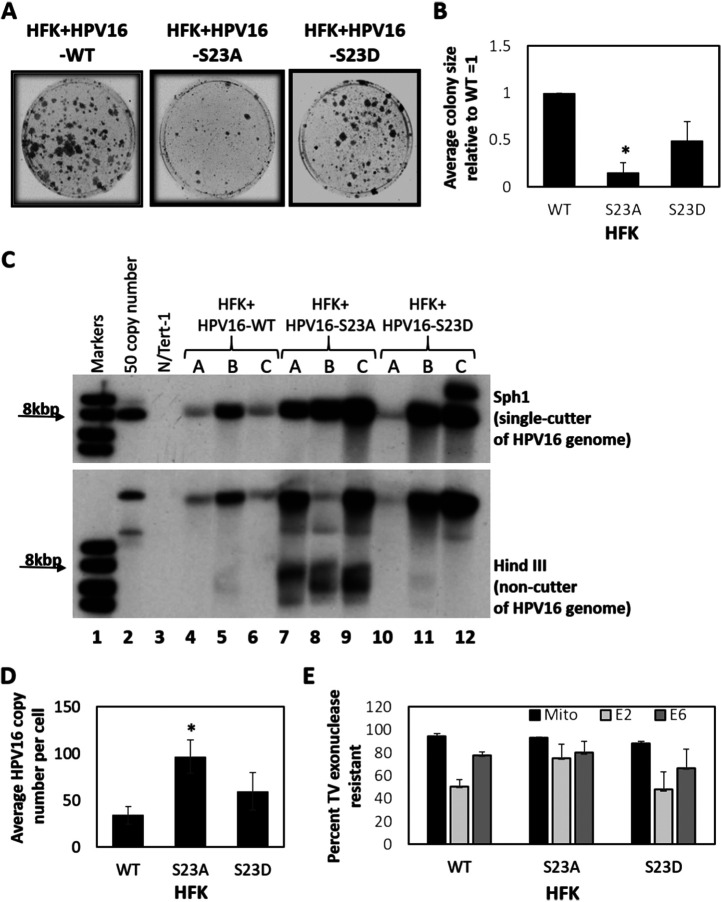
(A) Human foreskin keratinocytes (HFK) were transfected with the indicated HPV16 genomes, and cell colonies formed 2 weeks after transfection and selection with G418 (pcDNA3 G418-resistant plasmid colonies did not grow out, nor did nontransfected controls). There was a clear reduction in colony size in three independent HFK donors transfected with HPV16-S23A. This was quantitated, and the results are shown in panel B. The asterisk indicates a significant reduction in colony size for E2-S23A (*P* value < 0.05). (C) DNA extracted from the indicated cell lines were probed with the HPV16 genome in Southern blots. The top panel demonstrates the presence of 8-kbp bands in all samples following digestion with the HPV16 genome single cutter SphI. In the bottom panel, results with HindIII, which does not cut the HPV16 genome, are shown. In all cases, there is a higher-molecular-weight band that runs similarly to the control DNA (lane 2). (D) The bands in the top panel of panel C (SphI cut) were quantitated and are summarized here. The asterisk indicates a significant increase in HPV16-S23A genome copy number (*P* value < 0.05). (E) To investigate the episomal status of the HPV16 genomes in all cell lines, we used the TV exonuclease assay (see text for details). There was no significant difference in the episomal status of the HPV16 genomes between E2-WT, E2-S23A, or E2-S23D. Table S1 in the supplemental material details the results. Following establishment, the growth rates of the HFK+HPV16-WT, HFK+HPV16-S23A, and HFK+HPV16 S23D in monolayer cell culture were similar (Fig. S7).

To investigate the role of serine 23 during the HPV16 life cycle, we generated HPV16 genomes containing the E2 S23A and S23D mutations (HPV16-WT, HPV16-S23A, and HPV16-S23D). We transfected these genomes into three independent human foreskin keratinocyte (HFK) primary cell cultures to generate immortalized cell lines for life cycle studies. On the first attempt at immortalization, two out of three donors transfected with HPV16-WT and HPV16-S23D generated successful, immortalized cell lines, whereas none of the donors were successfully immortalized by the HPV16-S23A variant (not shown). In the second attempt, we optimized our immortalization procedure by including feeder cells during transfection and selection. Again, we got a striking phenotype with HFK+HPV16-S23A: all three keratinocyte cultures exhibited an attenuated initial immortalization, with slow growing colonies. Figure 6A shows an example for one of the HFK cultures 2 weeks following selection, crystal violet staining reveals the reduction in colony formation with HPV16-S23A compared with HPV16-WT and HPV16-S23D. Crystal violet staining following establishment was carried out in duplicate for all three lines, and the results are summarized in Fig. 6B.

10.1128/mBio.01163-21.7 FIG S7 The growth rates of the indicated cell lines were determined as described previously (97). There was no statistically significant difference between the growth of any of the cell lines. Download FIG S7, TIF file, 0.09 MB. Copyright © 2021 Prabhakar et al. 2021 Prabhakar et al. https://creativecommons.org/licenses/by/4.0/ This content is distributed under the terms of the Creative Commons Attribution 4.0 International license.

10.1128/mBio.01163-21.10 TABLE S1 Summary of results from the TV exonuclease assay described in the legend to Fig. 6. Download Table S1, XLSX file, 0.01 MB. Copyright © 2021 Prabhakar et al. 2021 Prabhakar et al. https://creativecommons.org/licenses/by/4.0/ This content is distributed under the terms of the Creative Commons Attribution 4.0 International license.

Even though initial immortalization was attenuated, HFK+HPV16-S23A cells eventually grew out successfully. Their growth rate was no different from HFK+HPV16-WT or HFK+HPV16-S23D (Fig. S7). To determine the status of the HPV16 genomes in the cells (episomal or integrated), Southern blot analysis was carried out (Fig. 6C). In all of the HPV16 lines, SphI (top blot) (cuts the HPV16 genome once) generated an 8-kbp signal. Lane 3 contained DNA from N/Tert-1 cells and generated no signal. A band of around 10 kbp was observed in HFK+HPV16-S23D-3. A HindIII (bottom blot) (does not cut the HPV16 genome) digest generated a slowly migrating species in all samples, indicative of open circular DNA. Cell lines from all three donors containing HPV16-S23A samples exhibit significantly faster migrating bands compared with cells containing WT and S23D genomes (compare lanes 7 to 9 with the others). A striking feature of the SphI digest is that there is more DNA present in the HFK lines containing the S23A variant compared with the WT (compare lanes 7 to 9 with lanes 4 to 6). The signals generated in the HPV16 lines were quantitated relative to the 50 copy number (lane 2) in the SphI digest. Figure 6D summarizes the quantitation; there is a statistically significant increase in HPV16 genome copy number in the S23A samples compared to the WT.

These HFK lines represent pools; therefore, there could be increased integration events in some of the lines compared with others. If there were a general level of increased integration, DNA would be integrated in millions of different sites and would not generate detectable signals on Southern blots. We used a recently developed technique that uses exonuclease-resistant DNA as a measure of episomal status to determine whether mutation of E2 S23 alters the episomal/integrated status of the HPV16 genomes (87–89). In this assay, DNA is treated with TV exonuclease (Exo) which degrades linear DNA, but not circular. We used glyceraldehyde-3-phosphate dehydrogenase (GAPDH) as our linear standard and designated the change in the threshold cycle (dCt) between samples plus and minus ExoV as 100% degradation. We then took the dCt for a mitochondrial marker (a circular genome) and E2 and E6 and determined the percentage of degradation by comparing the dCt difference with that of GAPDH. The data shown are a summary of the three cell lines generated (Fig. 6E). The circular mitochondrial DNA is around 90% resistant in all samples. E2 and E6 are between approximately 50 and 80% resistant. Table S1 in the supplemental material summarizes the results from these assays. Interestingly, HFK+HPV16-S23D-C is predominantly integrated, and the additional band on the Southern blot (Fig. 6C, lane 12) may be related to this. What is clear from this experiment is that there is not a significant difference between HFK+HPV16-WT and HFK+HPV16-S23A/S23D with regards to the episomal status of the viral genomes. Therefore, the introduction of these mutations does not promote integration of the viral genome into that of the host. To confirm that our assay robustly differentiates between episomal and integrated HPV16 genomes, we used W12 clone 20863 (which contains episomal HPV16 genomes) and W12 clone 20861 (which contains integrated HPV16 genomes). These clones were generated in the lab of Paul Lambert from the original cell line established from an HPV16-positive cervical lesion (90, 91). Figure S8A demonstrates that the HPV16 genome is degraded by TV exonuclease in clone 20861, but not in clone 20863, confirming the ability of this assay to differentiate between episomal and integrated HPV16 genomes.

**FIG 7:**
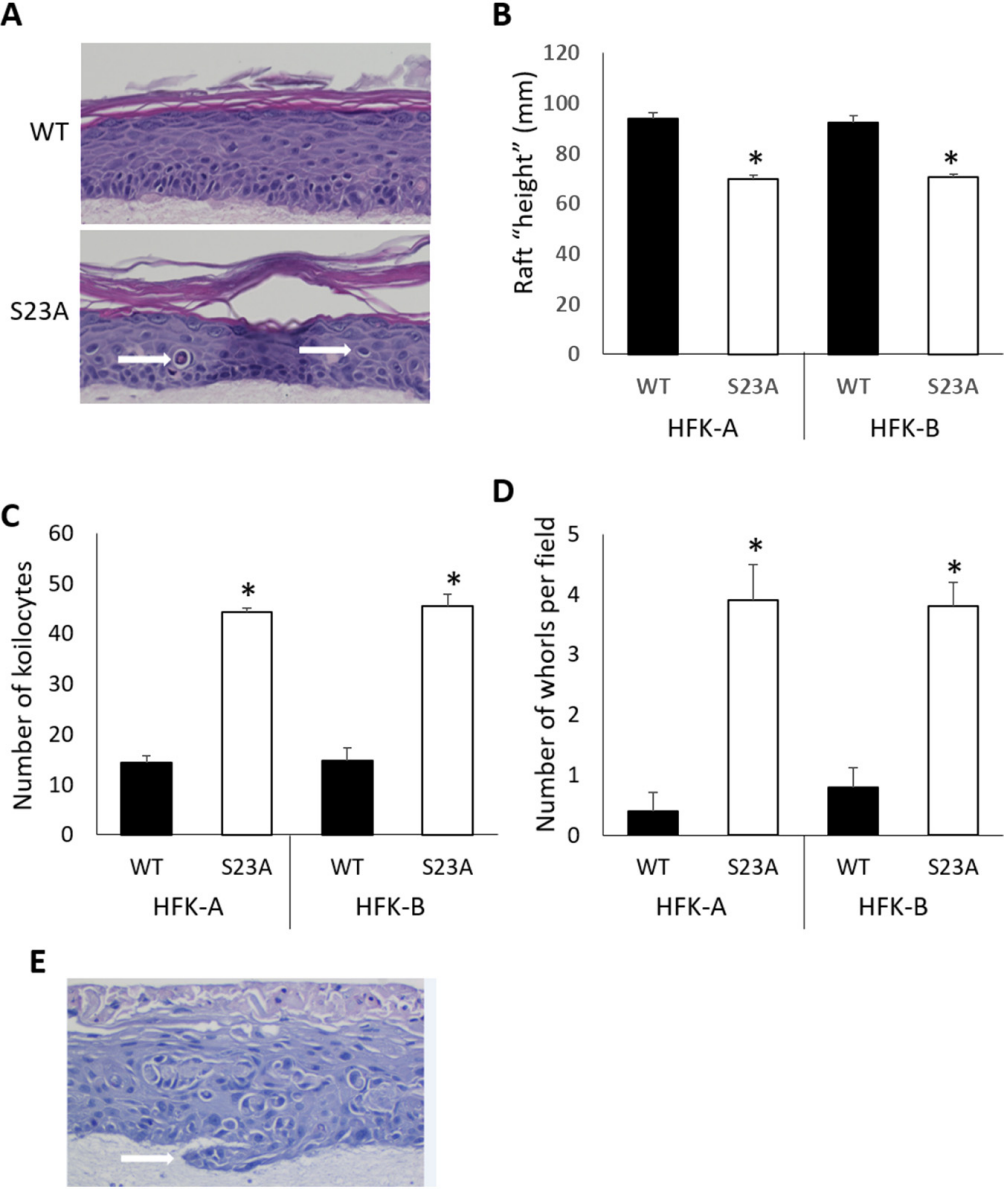
(A) Samples A and B from Fig. 6 were subjected to organotypic raft cultures, and H&E staining was carried out. The white arrows on the S23A panel point to koilocyte-like cells. (B) Duplicate rafts from two independent samples were scanned for their “height” using the Keyence imaging system, and the average height for each duplicate is shown. (C) The number of koilocytes in duplicate rafts from two independent samples was determined using the Keyence imaging system, and average numbers are shown. (D) The number of whorls observed in rafts from WT and S23A samples was determined in 10 independent images from each sample. The total number of whorls per raft was then determined. The asterisks in panels B to D indicate a *P* value of less than 0.05 for the difference between the WT and S23A samples. (E) Invasive keratinocytes were observed only in the S23A samples; an example is highlighted by a white arrow.

10.1128/mBio.01163-21.8 FIG S8 (A) To confirm that the TV exonuclease assay differentiates between episomal and integrated HPV16 genomes, we used clone W12 20863, which contains an episomal HPV16 genome (W12e), and clone W12 20861, which has an integrated HPV16 genome (W12i). DNA was prepared from three independent plates and subjected to the TV exonuclease assay as described in the main text. There is a highly significant reduction in E6 signal with TV in the W12i cells versus the W12e cells (*, *P* value < 0.05). This demonstrates that the assay is able to differentiate between cells containing HPV16 episomal or integrated HPV16 genomes. (B) A whorl observed more frequently in the organotypic raft cultures of HFK+HPV16-S23A than in HFK+HPV16-WT. See Fig. 7D for quantitation and statistical significance. Download FIG S8, TIF file, 1.6 MB. Copyright © 2021 Prabhakar et al. 2021 Prabhakar et al. https://creativecommons.org/licenses/by/4.0/ This content is distributed under the terms of the Creative Commons Attribution 4.0 International license.

While the growth of the cells was not different when grown on plastic, this does not represent a physiologically relevant part of the HPV16 life cycle. To investigate whether mutation of serine 23 disrupted the HPV16 life cycle, in addition to attenuating initial immortalization, we carried out organotypic raft cultures, as we have described previously (92, 93). Figure 7A gives a representative image from hematoxylin and eosin (H&E) staining of the organotypic rafts from donor A containing the wild-type (WT) HPV16 and HPV16 S23A genomes (Fig. 6). There is clearly a “thinner” epithelium with the HPV16 S23A genome containing cells. This is quantitated in Fig. 7B for two of the three HFK clones; the results are based on duplicate organotypic rafts for each donor. In addition, there appeared to be an increase in koilocytes with the mutant genomes (highlighted by the white arrows in Fig. 7A), and quantitation demonstrated this to be the case (Fig. 7C). Koilocytes are indicative of a more transformed cell, and in addition to these cells, we also observed a significant increase in whorls with the S23A mutant cells (see Fig. S8B for an example, and Fig. 7D for a quantitation). These whorls are found in HPV lesions (94), and HPV-positive ano-genital cancers and head and neck cancers (95, 96). In addition to the increased number of whorls in the S23A cells, we also observed several patches where the epithelial layer invades into the stromal collagen plug (Fig. 7E); this was never observed with the wild-type HPV16-containing cells.

**FIG 8:**
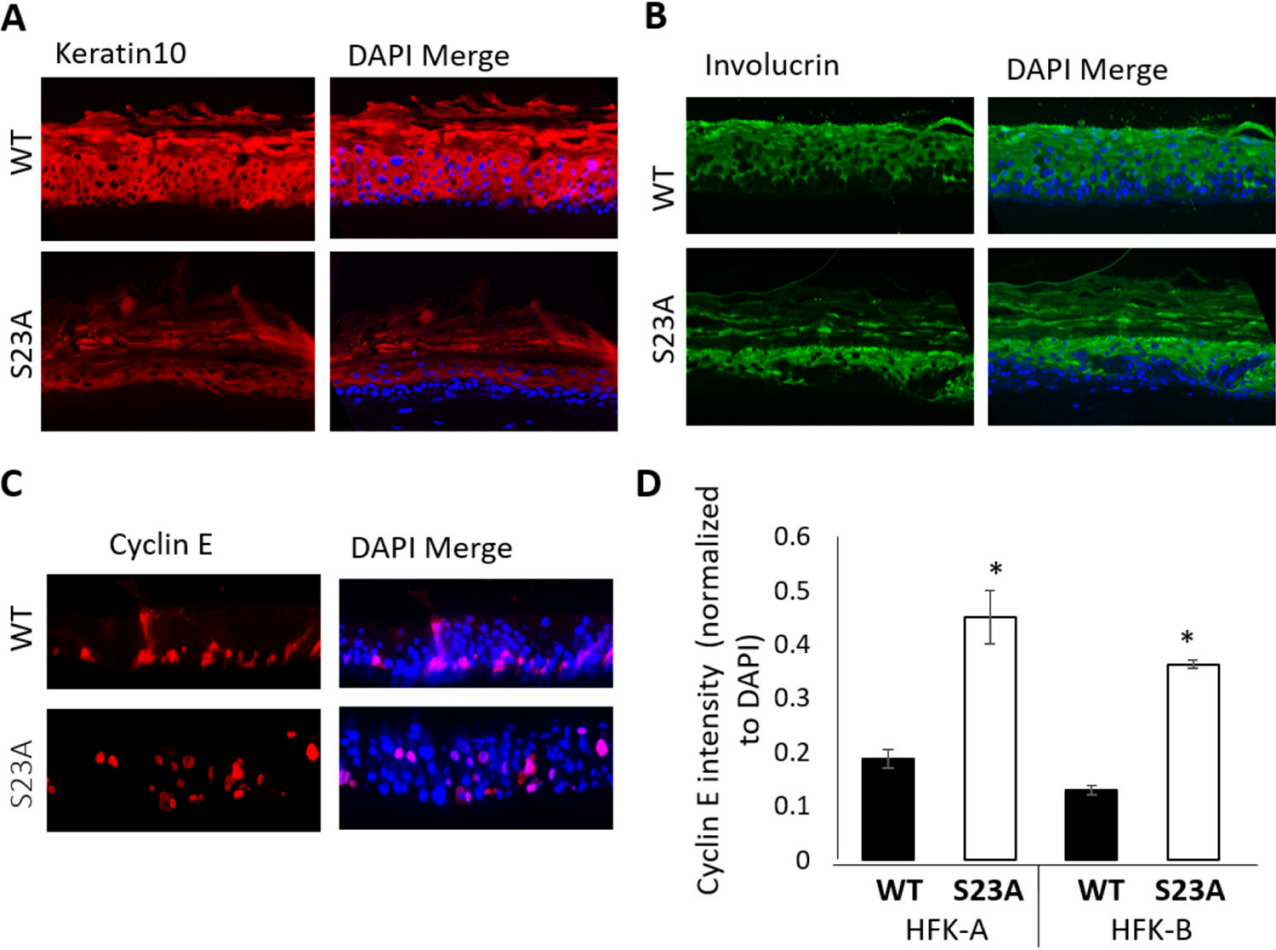
(A to C) Organotypic raft cultures were stained with the indicated antibodies (left panels), and right panels show a merge with DAPI for each of the antibodies. (D) A quantitation of cyclin E staining from two independent rafts of each donor sample. An asterisk indicates a *P* value of less than 0.05 for the difference between the WT and S23A samples.

The increased koilocytes, whorls, and invasive nature of the S23A cells prompted us to next investigate the differentiation status of the wild-type and S23A organotypic rafts. Figure 8A and B demonstrate keratin 10 and involucrin staining, respectively. Strikingly, the pattern of staining for both differentiation markers is different in the S23A cells compared with wild-type cells. Keratin 10 staining started “higher” in the epithelium in the S23A cells, while there are large patches of the S23A samples where there is an absence of involucrin staining reaching the upper layers of the epithelium. Both of these results suggest that the S23A cells are not differentiating correctly compared with the wild-type cells. To investigate the proliferative nature of the cells, we stained with cyclin E (Fig. 8C). Strikingly, there are many more cyclin E-positive cells in the upper layers of the S23A raft compared with the wild-type counterpart and more cyclin E positivity overall. This was quantitated in two independent rafts from two independent donors, and statistically significantly different levels of cyclin E2 were observed in the S23A cells (Fig. 8D).

**FIG 9:**
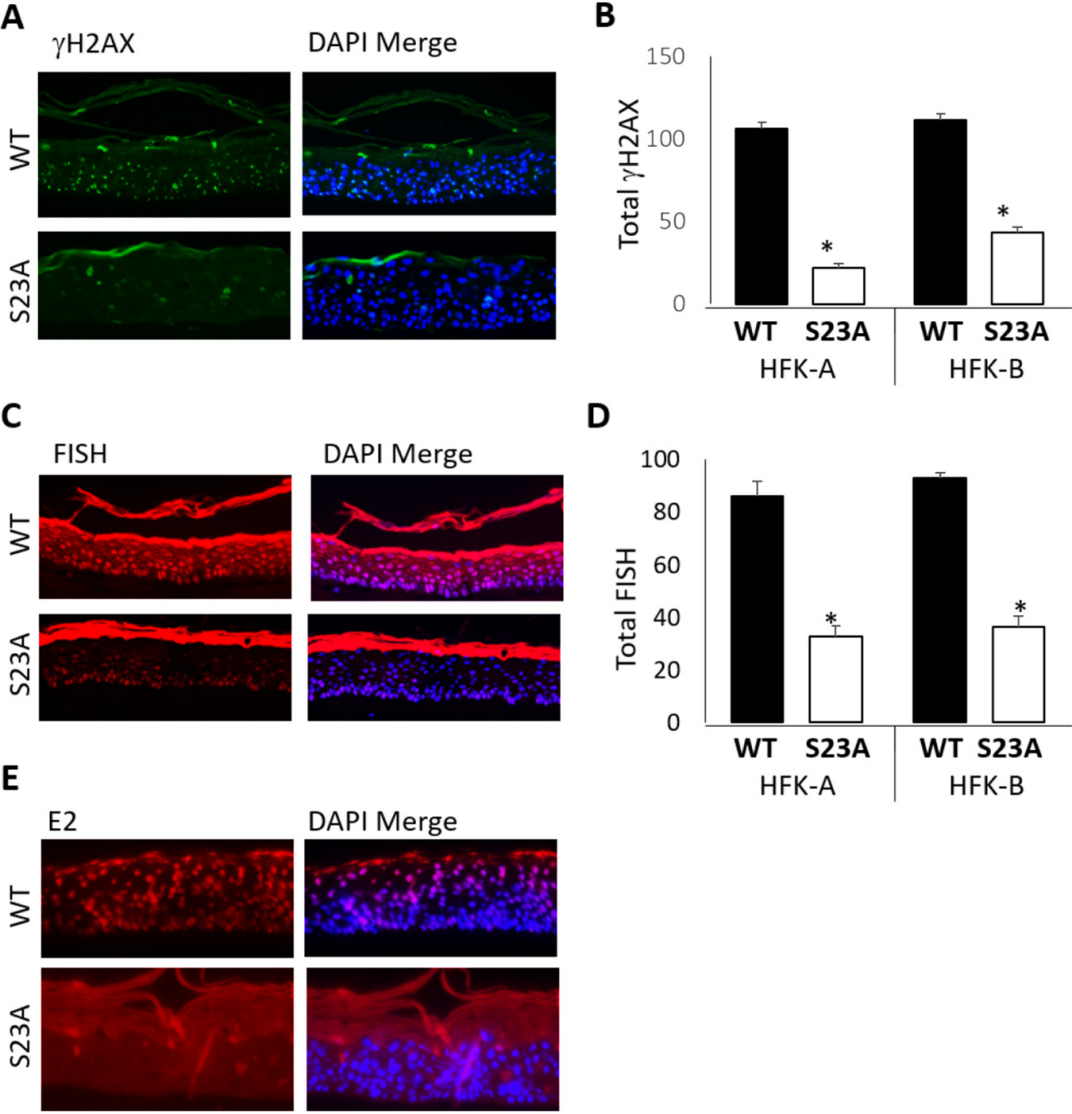
(A) γH2AX staining of the indicated rafts. The left-hand panels are antibody only; the right-hand panels are merged with DAPI. There is likely some autofluorescence with the keratin layers in the differentiated epithelium that are nonspecific. (B) A quantitation of the total gH2AX staining, determined using the Keyence imager system. (C) Fluorescent *in situ* hybridization (FISH) staining of the indicated rafts with a labeled HPV16 genome. Left-hand panels are FISH only, right-hand panels are merged with DAPI. There is a nonspecific interaction of the FISH probe with the keratin layers of the differentiated epithelium. (D) Quantitation of the DAPI layers for the FISH signal (therefore excluding the nonspecific interaction with the keratin layer) using the Keyence imager system. An asterisk in panel B and D indicates a *P* value of less than 0.05 for the difference between the WT and S23A samples. (E) E2 staining of the indicated rafts. The left-hand panels are antibody only; the right-hand panels are merged with DAPI. HPV16 E2 B9 monoclonal antibody was used in Fig. 9E.

The results in Fig. 7 and 8 demonstrate an aberrant epithelium with the S23A cells compared with the wild type. We next investigated markers of the viral life cycle. Figure 9A demonstrates a lack of γH2AX staining in the S23A tissues that was statistically significantly reduced in two independent donor samples (Fig. 9B). γH2AX is a marker of viral replication in organotypic raft cultures, as viral replication induces replication stress and activates the DNA damage response (69, 97). The results therefore suggested that there was a failure of viral replication in the S23A cells, and to further investigate this, we carried out FISH staining with labeled HPV16 genome to determine the levels of viral DNA in the rafts (Fig. 9C). Agreeing with the γH2AX staining, there is a loss of viral genomes in the S23A samples as the cells migrate upwards through the epithelium. This was statistically significant in two independent donors (Fig. 9D). The loss of staining indicates that the viral genome is likely not being replicated; therefore, the viral genomes are being “diluted” as the cells proliferate and differentiate. Even in the basal layers, it is noticeable that there is less signal with the S23A samples compared to the wild-type cells. There is more viral DNA in the S23A cells when they are grown on plastic (Fig. 6C and D); therefore, the results suggest that there is an immediate failure to replicate the viral genome upon interaction of the cells with the stromal collagen/fibroblast plug. Upon seeding onto the collagen plugs, the cells divide several times before they are induced to differentiate, indicating that the S23A cells are not replicating viral DNA. Given that the E2-TopBP1 interaction stabilizes the E2 protein during mitosis (Fig. 5), we investigate the expression of E2 in the S23A and wild-type rafts (Fig. 9E; this is representative of two independent donor lines). There is clear nuclear E2 staining detectable in the wild-type cells throughout the epithelium. In the upper layers of the epithelium, there is an apparent increase in E2 staining intensity. However, with the S23A sample, there is a loss of E2 nuclear staining, although there is a background signal due to nonspecific interaction with the keratin (that has been “stripped” from the wild-type sample during processing). Therefore, there may be some residual E2 expression in the S23A sample, but it is dramatically reduced.

## DISCUSSION

Here we demonstrate that CK2 phosphorylation of E2 serine 23 results in complex formation with TopBP1 *in vitro* and *in vivo*. The conservation of serine 23 across α-HPV types (Fig. 1), and the negative aspartic and glutamic acid residues at positions −1 and −3, respectively, indicate a potential CK2 target residue (76). *In vivo*, mutation of S23 to alanine disrupts the coimmunoprecipitation of E2 with TopBP1, while an aspartic acid mutation (giving a negative charge mimicking phosphorylation) retains interaction (Fig. 1). To demonstrate phosphorylation of S23 *in vivo*, we generated a phospho-specific antibody (pS23-Ab) which recognizes E2-WT, including during mitosis, but not E2-S23A (Fig. 3). CK2 functions as a tetramer with two β-subunits and two α- or α′-subunits, the latter being the enzymatic components of the complex (76). In Fig. 3, knockdown of either α component, or partial knockdown of both, abolished detectable levels of E2 S23 phosphorylation in U2OS cells and partially disrupted the interaction between E2 and TopBP1. The reason for the complete loss of phosphorylation, and only a partial loss of interaction, could be due to the failure to detect residual E2 phosphorylation following knockdown of the CK2 components. Unfortunately, pS23-Ab did not work on Western blots; it is possible the antibody recognizes only native E2 and not the denatured versions generated for Western blotting. Another result supporting the important role for CK2 in the phosphorylation of S23 in U2OS cells is that addition of the CK2 inhibitor CX4945 abolished detectable phosphorylation on this residue (as determined by pS23-Ab immunoprecipitation) and also disrupted the E2-TopBP1 interaction (Fig. 3). We extended our studies to demonstrate that S23 is critical for the E2-TopBP1 interaction in N/Tert-1 cells and that CX4945 abolishes detectable phosphorylation of E2 on this residue and blocks the E2-TopBP1 interaction in N/Tert-1 cells. We also observed detectable E2 S23 phosphorylation during the HPV16 life cycle in N/Tert-1 cells (Fig. 4). CK2 was expressed in HPV16-positive cells but was reduced compared with control cells indicating that HPV16 perhaps regulates CK2 levels during the differentiation process (Fig. 4E).

As well as demonstrating that CK2 phosphorylation of S23 mediates the E2-TopBP1 interaction *in vivo*, we also demonstrated that CK2 controls this interaction *in vitro* (Fig. 2). E2-WT cannot interact with TopBP1 *in vitro*, while E2-S23D can. Incubation of E2-WT with CK2 promotes the interaction between E2 and TopBP1 recombinant proteins, and this can be reversed by treatment with lambda phosphatase. As E2 has been shown to interact with CK2 components (78), the latter treatment is important, as it demonstrates that the enzymatic function of CK2 is required to promote the E2-TopBP1 interaction and that it does not act as a “bridge” to bring the two proteins together. The combination of *in vivo* and *in vitro* results demonstrates that CK2 phosphorylation of E2 S23 is crucial for E2-TopBP1 complex formation.

Previous studies have identified several TopBP1 domains that interact with phosphorylated peptides (80). The region around E2 S23 does not correspond to a consensus sequence for interacting with any of these TopBP1 domains, and indeed, an E2 pS23 peptide does not interact with any of these domains (see Fig. S9 in the supplemental material). This indicates that E2 interacts with a yet to be determined domain of TopBP1. Future studies will identify this domain; it will be interesting to determine whether E2 has evolved a unique way to interact with TopBP1 that does not disrupt the ability of TopBP1 to interact with host proteins involved in the DNA damage response, a process important for the HPV life cycle (69).

10.1128/mBio.01163-21.9 FIG S9 The E2 pS23 peptide does not interact with well-characterized phospho-peptide binding domains of TopBP1. Fluorescent polarization assays were carried out with the indicated peptides and TopBP1 BRCT-containing domains. The control peptides are known interactors of the indicated TopBP1 domains (see reference 80 for details). Download FIG S9, TIF file, 0.1 MB. Copyright © 2021 Prabhakar et al. 2021 Prabhakar et al. https://creativecommons.org/licenses/by/4.0/ This content is distributed under the terms of the Creative Commons Attribution 4.0 International license.

Previous studies have implicated CK2 in several aspects of papillomavirus functions. CK2 phosphorylation of bovine papillomavirus 1 (BPV1) E2 on 301 regulates the stability of this protein (98), although this residue is not conserved on HPV16 E2 and both E2-WT and E2-S23A are expressed at relatively equivalent levels in both U2OS and N/Tert-1 cells. CK2 can regulate the DNA binding of BPV and HPV E1 proteins and can control their DNA replication functions (99). CK2 phosphorylates and regulates HPV18 E1 function and is important in the life cycle of HPV18 and -11 (86, 100). CK2α was the critical component involved in regulating E1, CK2α′ was not involved. CK2 phosphorylation of BRD4 is important for mediating HPV16 E2 transcription and replication function, and we and others have demonstrated that a direct interaction between E2 and BRD4 is required for E2 transcription function (34, 101, 102). As well as regulating E1-E2 functions, CK2 can also regulate the function of E7 proteins. Phosphorylation of a CK2 consensus sequence on E7 is important for E7 degradation of p130 and the promotion of S phase in differentiated keratinocytes (103), and a HPV18 E7 CK2 target residue is required for maintaining the transformed phenotype of cervical cancer cells (104).

In transient replication and transcription assays, the E2-S23A function is similar to E2-WT (Fig. S3). A third major function for E2 in the viral life cycle is to actively associate with viral and human DNA simultaneously during mitosis; it is proposed that this function results in segregation of the viral genomes into daughter nuclei following mitosis (25). E2 has been shown to bind to mitotic chromatin, and previously we demonstrated costaining of E2 and TopBP1 on mitotic chromatin (37). Therefore, we investigated the interaction of E2-WT and E2-S23A with mitotic chromatin (Fig. 5). E2-WT showed robust staining on mitotic chromatin, and in addition, it recruited TopBP1 onto the mitotic chromatin. In control cells with no E2, TopBP1 does not “coat” the mitotic chromatin as it does with E2-WT. Therefore, like BPV1 E2 and BRD4, E2 alters TopBP1 interaction with mitotic chromatin (29). For E2-S23A, there was a reproducible reduction in E2 staining on mitotic chromatin although it was located on the mitotic chromatin, and also recruited TopBP1 to the mitotic chromatin. Cell cycle analysis demonstrates that E2-WT levels are increased during mitosis, while E2-S23A levels are not, agreeing with the staining patterns observed. Additionally, E2-WT increases the levels of TopBP1 during mitosis, while E2-S23A cannot. Therefore, E2-WT and E2-S23A have distinct phenotypes during mitosis. We propose that there is an additional factor that mediates the interaction of E2-S23A with mitotic chromatin, and this is under active investigation. BRD4 is clearly a leading candidate we are focusing on.

Introduction of the S23A mutation into the HPV16 genome resulted in a delay in immortalization, although the resulting cells that grew out retained episomal viral genomes (Fig. 6). These cells were grown on plastic with no feeder cells, and the S23A mutation had no effect on cell proliferation when the cells were grown in this manner. However, this is not reflective of any aspect of the HPV16 life cycle, and to determine the effect of the S23A mutation on this process, we submitted cells to organotypic rafting. This resulted in several striking phenotypes, indicating an epithelial-stroma interaction that preferentially affects the HPV16 S23A-containing cells; there is a complex interaction between HPV-infected cells and the stroma (105). Morphologically, the S23A cells looked more dysplastic than the wild-type cells with a “thinner” epithelium; there was an increase in the number of koilocytes and whorls as well as detection of epithelial invasion into the collagen/fibroblast stroma (Fig. 7). The S23A cells were also less differentiated and more proliferative than wild-type cells, again supporting the idea of an aberrant life cycle and increased dysplasia with the S23A mutant genome cells. We demonstrate that there is a failure of viral replication with the S23A mutant resulting in a reduction in viral genomes in the mid-to-upper layers of the differentiated raft (Fig. 9A to D). Finally, we demonstrate that there is a dramatic loss of E2 expression in the S23A cells compared with the wild-type cells (Fig. 9E). The dramatic effects of the S23A mutation during organotypic rafting compared with cells cultured on plastic could be related to the interaction of the HPV16-positive cells with the collagen/fibroblast stroma and/or to the differentiation status of the cells. We are currently investigating these possibilities.

TopBP1 is involved in active replication during mitosis (45, 47, 71, 73, 106). A model to explain the S23A phenotype is that the E2-TopBP1 interaction locates the viral genome to sites on chromatin that allow replication of the viral genome during mitosis or to sites that will promote viral replication during the subsequent S phase following mitosis. Another model is that the E2-TopBP1 interaction is required for the stabilization of E2 in the differentiating epithelium and that without E2 the viral genome will not be replicated. It is also possible that the S23A mutant is somehow promoting integration of the viral genome into the host due to an aberrant viral replication function resulting in a loss of E2 expression. We do not favor the latter idea as it appears the viral genome does get “diluted” during differentiation, suggesting it has not all integrated (see the fluorescent *in situ* hybridization [FISH] staining in Fig. 9C). However, integration of a subset of viral genomes with the S23A mutant would potentially explain the more transformed phenotypes of the S23A cells, as integration is associated with a more aggressive HPV-positive tumor. The lack of detectable E2 expression could also disrupt E2 genome segregation during mitosis, which would also result in a “dilution” of the viral genomes. However, due to the failure to detect γH2AX (as a surrogate marker for viral replication), we favor that E2 degradation and a failure to replicate causes the phenotypes we observe with the S23A genome-containing cells. It is of course possible that multiple aberrant functions of E2 S23A could contribute to the aberrant life cycle.

Future work will focus on understanding the mechanism of the aberrant life cycle promoted by the S23A mutant. This report demonstrates that the E2-TopBP1 interaction is critical for the HPV16 life cycle and that disrupting it could potentially promote oncogenesis. Therefore, the E2-TopBP1 interaction may act as a “tumor suppressor complex” to control the oncogenic properties of HPV16. The results presented also caution against using CK2 inhibitors for the treatment of HPV16 infections. While such inhibitors would abolish viral production and therefore potentially block viral transmission, they could also promote the more transformed phenotypes observed with the S23A genomes. This could therefore promote oncogenic progression of HPV16 lesions. As the CK2 inhibitor CX4945 is currently in anticancer combination therapy clinical trials, it will be important for future studies to determine whether such inhibitors do promote HPV-related lesions/cancers in treated individuals (107).

## MATERIALS AND METHODS

### Generation and culture of stable cell lines.

Stable cell lines expressing wild-type E2 (E2-WT), E2-S23A, and E2-S23D, along with pcDNA empty vector plasmid control were established both in U2OS and N/Tert-1 cell lines as previously described (23, 24). Cell culture was also performed as described in these publications.

### Western blotting.

Protein from cell pellets was extracted with 2× pellet volume protein lysis buffer (0.5% Nonidet P-40, 50 mM Tris [pH 7.8], and 150 mM NaCl) supplemented with protease inhibitor (Roche Molecular Biochemicals) and phosphatase inhibitor cocktail (Sigma). The cells were lysed for 20 min on ice followed by centrifugation at 18,000 rcf (relative centrifugal force) for 20 min at 4°C. Protein concentration was estimated colorimetrically using a Bio-Rad protein assay. Fifty micrograms of protein with equal volume of 4× Laemmli sample buffer (Bio-Rad) was denatured at 95°C for 5 min. The samples were run on a Novex WedgeWell 4% to 12% Tris-glycine gel (Invitrogen) and transferred onto a nitrocellulose membrane (Bio-Rad) using the wet-blot method, at 30 V overnight. The membrane was blocked with Li-Cor Odyssey blocking buffer (phosphate-buffered saline [PBS]) diluted 1:1 (vol/vol) with PBS and then incubated with the specified primary antibody in Li-Cor Odyssey blocking buffer (PBS) diluted 1:1 with PBS. Following this, the membrane was washed with PBS supplemented with 0.1% Tween 20 (PBS-Tween) and further probed with the Odyssey secondary antibodies (IRDye 680RD goat anti-rabbit IgG [H+L] [0.1 mg] or IRDye 800CW goat anti-mouse IgG [H+L] [0.1 mg]) in Li-Cor Odyssey blocking buffer (PBS) diluted 1:1 with PBS at 1:10,000 for 1 h at room temperature. After washing with PBS-Tween, the membrane was imaged using the Odyssey CLx Imaging System, and ImageJ was used for quantification. Primary antibodies used for Western blotting studies are as follows: HPV16 E2 (TVG 261) or monoclonal B9 (1:500) (Abcam ab17185 for TVG261 [108] for monoclonal B9), TopBP1 (1:1,000) (catalog no. A300-111A; Bethyl), glyceraldehyde-3-phosphate dehydrogenase (GAPDH) (1:10,000) (catalog no. sc-47724; Santa Cruz), casein kinase IIα (1AD9) (1:500) (catalog no. sc-12738; Santa Cruz), CKII alpha' antibody (1:1,000) (catalog no. A300-199A; Bethyl).

### Immunoprecipitation.

Primary antibody of interest or a HA tag antibody (used as a negative control) was incubated in 250 μg of cell lysate (prepared as described above), made up to a total volume of 500 μl with lysis buffer (0.5% Nonidet P-40, 50 mM Tris [pH 7.8], and 150 mM NaCl), supplemented with protease inhibitor (Roche Molecular Biochemicals) and phosphatase inhibitor cocktail (Sigma) and rotated at 4°C overnight. The following day, 40 μl of prewashed protein A beads per sample (Sigma; equilibrated to lysis buffer as mentioned in the manufacturer’s protocol) was added to the lysate-antibody mixture and rotated for another 4 h at 4°C. The samples were gently washed with 500 μl lysis buffer by centrifugation at 1,000 rcf for 2 to 3 min. This wash was repeated four times. The bead pellet was resuspended in 4× Laemmli sample buffer (Bio-Rad), heat denatured, and centrifuged at 1,000 rcf for 2 to 3 min. Proteins were separated using a sodium dodecyl sulfate-polyacrylamide gel electrophoresis (SDS-PAGE) system and transferred onto a nitrocellulose membrane before probing for the presence of E2 or TopBP1, as per the Western blotting protocol.

### Immunofluorescence and cell synchronization.

U2OS cells expressing stable E2-WT, E2-S23A, and pcDNA empty vector plasmid control were plated on acid-washed, poly-l-lysine-coated coverslips, in a six-well plate at a density of 2 × 10^5^ cells/well (5 ml Dulbecco modified Eagle medium [DMEM] plus 10% fetal bovine serum [FBS] [DMEM-FBS]). After 24 h, the cells were treated with 2 mM thymidine diluted in the DMEM-FBS for 16 h. This was then washed two times with PBS and recovered in supplemented DMEM. After 8 h, to block the cells at G_1_/S phase, a second dose of 2 mM thymidine was added and incubated for 17 h. The cells were then washed twice with PBS and recovered as before for 3 h. The cells were next treated with nocodazole (100 ng/ml) for 5 h and released for 2 h to enrich for mitotic cells. Following this, the cells were washed twice with PBS, fixed, and stained as described in reference 34. The primary antibodies used are as follows: HPV16 E2 (TVG 261) (1:500) (Abcam; ab17185), HPV16 E2 B9 monoclonal antibody (1:500) (108), TopBP1 (1:1,000) (catalog no. A300-111A; Bethyl), pS23-Ab (1:10,000) (custom generated by GenScript; peptide sequence, CKILTHYENDS^P^TDLR). The cells were washed and incubated with secondary antibodies Alexa Fluor 488-labeled goat anti-mouse (catalog no. A-11001; Thermo Fisher) and Alexa Fluor 594-labeled goat anti-rabbit (catalog no. A-11037; Thermo Fisher) diluted 1:1,000. The wash step was repeated, and the coverslips were mounted on a glass slide using Vectashield mounting medium containing 4′,6′-diamidino-2-phenylindole (DAPI). Images were captured with a Zeiss LSM700 laser scanning confocal microscope and analyzed using Zen LE software.

### Cell synchronization and Western blotting.

U2OS cells expressing stable E2-WT, E2-S23A, and pcDNA empty vector plasmid control were plated at 3 × 10^5^ density onto 100-mm plates in DMEM plus 10% FBS. The cells were treated with 2 mM thymidine diluted in the supplemented DMEM for 16 h. The cells were then washed two times with PBS and recovered in supplemented DMEM. After 8 h, to block the cells at G_1_/S phase, a second dose of 2 mM thymidine was added and incubated for 17 h. The cells were then washed twice with PBS and recovered as before at the following time points: 0 h and 2 h (G_1_/S phase), 4 h and 6 h (S phase), 8 h (M1 phase), 10 h (M2 phase), and 12 h (the next G_1_ phase). The cell lysate was prepared using the harvested cells at different time points, and immunoblotting was carried out as described above.

### Small interfering RNA (siRNA) and segregation assay.

U2OS parental cells were plated on 100-mm plates. The next day, cells were transfected with 10 μM following siRNA. 10 μM MISSION siRNA Universal Negative Control (catalog no. SIC001; Sigma-Aldrich) was used as a “nontargeting” control in our experiments. Lipofectamine RNAiMAX transfection (catalog no. 13778-100; Invitrogen) protocol was used in the siRNA knockdown. Forty-eight hours posttransfection, the cells were harvested, and knockdown was confirmed by immunoblotting for the protein of interest. CK2a siRNA was GGCUCGAAUGGGUUCAUCUtt (Sigma-Aldrich). CK2a′ siRNA was CAGUCUGAGGAGCCGCGAGdTdT.

### Production of recombinant protein.

Amino acids (aa) 1 to 200 of E2-WT and E2-S23A were produced as a fused protein with His tag, and TopBP1 was produced as a fused protein with a glutathione *S*-transferase (GST) tag (GST TopBP1 [aa 32 to 1522] His from Addgene; plasmid 20375). The protein expression was carried out by picking a single colony of Escherichia coli BL21(DE3) competent (catalog no. C2527; NEB Inc.) and growing it in LB medium supplemented with 100 μg/ml of selective antibiotics (kanamycin for His-tagged E2-WT and E2-S23D; ampicillin for GST-tagged TopBP1), grown overnight at 37°C, and shaken at a low speed. This starter culture was then diluted 1:100 in fresh LB medium with kanamycin. The culture was shaken at 37°C until the optimal density of 0.6 to 0.8 at an optical density at 600 nm (OD_600_) was achieved. Following this, isopropyl-β-d-thiogalactopyranoside (IPTG) at a final concentration of 1 mM was added to the culture, for induction of protein expression, shaking at 16°C overnight. His-tagged proteins were purified on nickel-nitrilotriacetic acid (Ni-NTA agarose) (catalog no. 30761; Qiagen), and GST-tagged TopBP1 protein was purified on Glutathione Sepharose 4B (catalog no. 17-0756; GE Health Care), according to the batch purification method described in the manufacturer’s manual, followed by size exclusion chromatography. The purity of the recombinant protein was confirmed by SDS-PAGE analysis.

### *In vitro* GST pulldown assays.

Purified recombinant His-tagged E2-WT and E2-S23D protein and GST-tagged TopBP1 were used for the *in vitro* pulldown assays. GST-tagged NEDD4 E3 ligase was used as our GST control. GST-TopBP1 and GST control were kept stable at 0.65 pmol, and 11 pmol of His-E2-WT and His-E2-S23D was used for the experiment. Glutathione Sepharose 4B (catalog no. 17-0756; GE Health Care), equilibrated to the GST lysis buffer (50 mM Tris-HCl [pH 8.0], 100 mM NaCl, 0.5 mM EDTA, 0.5 mM EGTA, 0.5% NP-40, 1 mM dithiothreitol [DTT] plus protease inhibitors) was added, and each tube was placed at 4°C for 1 h with continual end-to-end rotation. The protein-bound GST beads were washed three times in the GST lysis buffer by centrifugation at 1,000 rcf for 3 min and resuspended in 4× Laemmli sample buffer (Bio-Rad), heat denatured, and centrifuged at 1,000 rcf for 3 min. The supernatant was gel electrophoresed using an SDS-PAGE system which was later transferred onto a nitrocellulose membrane using wet-blot transfer method. The membrane was probed for the presence of E2 or TopBP1 as described above.

### *In vitro* kinase assay with or without lambda phosphatase.

Immunoprecipitated GST beads were prepared as mentioned above in the GST pulldown section. After 1 h, the beads were incubated with 1 μl CK2 enzyme and 1× CK2 reaction buffer (catalog no. P6010S; NEB Inc.) supplemented with 200 μM ATP and 30 mM MgCl_2_ and rotated for 1 h at 30°C. The beads were then incubated in the presence or absence of lambda phosphatase (catalog no. sc-200312A; Santa Cruz) as described in the manufacturer’s protocol. Following this, the beads were washed and analyzed by immunoblotting.

### CK2 inhibitor treatment.

U2OS and N/Tert-1 cells were plated at a density of 2 × 10^5^ in a 100-mm plate. The next day, the cells were treated with 10 μM CK2 inhibitor, CX-4945 (Silmitasertib) from APExBIO (catalog no. A8330) or 10 μM dimethyl sulfoxide (DMSO) for 48 h. The cells were then harvested and processed for immunoprecipitation with pS23Ab or TopBP1 as described.

### Immortalization of human foreskin keratinocytes (HFK).

HPV16 mutant genomes (S23A and S23D) were generated by Genscript. The HPV16 (WT, S23A, S23D) were removed from their parental plasmid using SphI, and the viral genomes were isolated and then recircularized using T4 ligase (NEB) and transfected into early passage HFK from three donor backgrounds (Lifeline Technology), alongside a G418 resistance plasmid, pcDNA. Cells underwent selection in 200 μg/ml G418 (Sigma-Aldrich) for 14 days and were cultured on a layer of J2 3T3 fibroblast feeders (NIH), which had been pretreated with 8 μg/ml mitomycin C (Roche). Throughout the immortalization process, HFK were cultured in Dermalife-K complete medium (Lifeline Technology). In Fig. 5A, transfected cells were stained with crystal violet 14 days following transfection and selection prior to passaging.

### Southern blotting.

Total cellular DNA was extracted by proteinase K-sodium dodecyl sulfate digestion followed by a phenol-chloroform extraction method. Five micrograms of total cellular DNA was digested with either SphI (to linearize the HPV16 genome) or HindIII (which does not cut the HPV16 genome). All digests included DpnI to ensure that all input DNA was digested and not represented as replicating viral DNA. All restriction enzymes were purchased from NEB and utilized as per manufacturer’s instructions. Digested DNA was separated by electrophoresis of a 0.8% agarose gel, transferred to a nitrocellulose membrane, and probed with radiolabeled (^32^P) HPV16 genome. This was then visualized by exposure to film for 1 to 24 h. Images were captured from an overnight-exposed phosphor screen by GE Typhoon 9410 and quantified using ImageJ.

### Exonuclease V assay.

To examine whether viral genomes were maintained as episomes, we carried out an exonuclease V assay, as described by Bienkowska-Haba et al. (89), which determines the resistance of HPV16 genomes to exonuclease V. Twenty nanograms of genomic DNA was either treated with exonuclease V (RecBCD, NEB), in a total volume of 30 μl, or left untreated for 1 h at 37°C followed by heat inactivation at 95°C for 10 min. Two nanograms of digested/undigested DNA was then quantified by real-time PCR using a 7500 FAST Applied Biosystems thermocycler with SYBR green PCR Master Mix (Applied Biosystems) and 100 nM each primer in a 20-μl reaction mixture. Nuclease-free water was used in place of the template for a negative control. The following cycling conditions were used: 50°C for 2 min, 95°C for 10 min, 40 cycles at 95°C for 15 s, and a dissociation stage of 95°C for 15 s, 60°C for 1 min, 95°C for 15 s, and 60°C for 15 s. Separate PCRs were performed to amplify HPV16 E6 F (forward), 5′- TTGCTTTTCGGGATTTATGC-3′, and R (reverse), 5′-CAGGACACAGTGGCTTTTGA-3′; HPV16 E2 F, 5′-TGGAAGTGCAGTTTGATGGA-3′, and R, 5′-CCGCATGAACTTCCCATACT-3′; human mitochondrial DNA F, 5′-CAGGAGTAGGAGAGAGGGAGGTAAG-3′, and R, 5′-TACCCATCATAATCGGAGGCTTTGG-3′; and human GAPDH DNA F, 5′-GGAGCGAGATCCCTCCAAAAT-3′, and R, 5′-GGCTGTTGTCATACTTCTCATGG-3′.

### Organotypic raft culture.

Keratinocytes were differentiated via organotypic raft culture as described previously (81, 93). Briefly, cells were seeded onto type 1 collagen matrices containing J2 3T3 fibroblast feeder cells. Cells were then grown to confluence atop the collagen matrices, lifted onto wire grids, and cultured in cell culture dishes at the air-liquid interface, with medium replacement on alternate days. Following 13 days of culture, rafted samples were fixed with formaldehyde (4% [vol/vol]) and embedded in paraffin blocks. Multiple 4-μm sections were cut from each sample. Sections were stained with hematoxylin and eosin (H&E), and other sections were prepared for immunofluorescent staining via HIER. Fixing and embedding services in support of the research project were generated by the VCU Massey Cancer Center Cancer Mouse Model Shared Resource. Fixed sections were antigen retrieved in citrate buffer and probed with the following antibodies for immunofluorescence analysis: phospho-γH2AX (1/500) (9718; Cell Signaling Technology), cyclin E (1/1,000) (sc-247; Santa Cruz Biotechnology), involucrin (1/1,000) (ab27495; abcam), keratin 10 (1/1,000) (SAB4501656; SigmaAldrich), CK2α (1/1,000) (SC1273). Cellular DNA was stained with 4′,6′-diamidino-2-phenylindole (DAPI) (sc-3598; Santa Cruz). Fluorescent *in situ* hybridization (FISH) staining for HPV16 genomes was performed using Alexa Fluor 594-labeled HPV16 genomes, generated using the Alexa Fluor 594 DNA labeling kit (Thermo Fisher) as per manufacturer’s instructions. Microscopy and subsequent image analysis (percent staining and staining intensity) was performed using the Keyence imaging system, whereby whole stained sections were scanned computationally and the fluorescence intensity was calculated compared to a negative background control (secondary antibody only) and a positive localization control (DAPI). Intensity was calculated based on the number of photons at a specific location, thus determining the local concentration of fluorophores (secondary antibodies). In this way, this is equivalent to measuring densitometry to estimate protein concentration from Western blots. The same imaging parameters were used for each slide and for each sample, and two sections from two individually grown rafts were scanned to generate average values. Immunofluorescence was observed using a Keyence BZ-X800 microscope and analyzed using BZ-X800 Analyzer software (Keyence Corporation of America).

### Statistical analysis.

All the data are represented as means ± standard errors (SE). Significance was determined using a Student’s *t* test, and standard error was calculated from independent experiments.
