# Supplementary figures and images for: CK2 Phosphorylation of Human Papillomavirus 16 E2 on Serine 23 Promotes Interaction with TopBP1 and Is Critical for E2 Interaction with Mitotic Chromatin and the Viral Life Cycle
Source: mBio. 2021 Sep 21;12(5):e01163-21. doi: 10.1128/mBio.01163-21 (PMC8546539; doi:10.1128/mBio.01163-21)

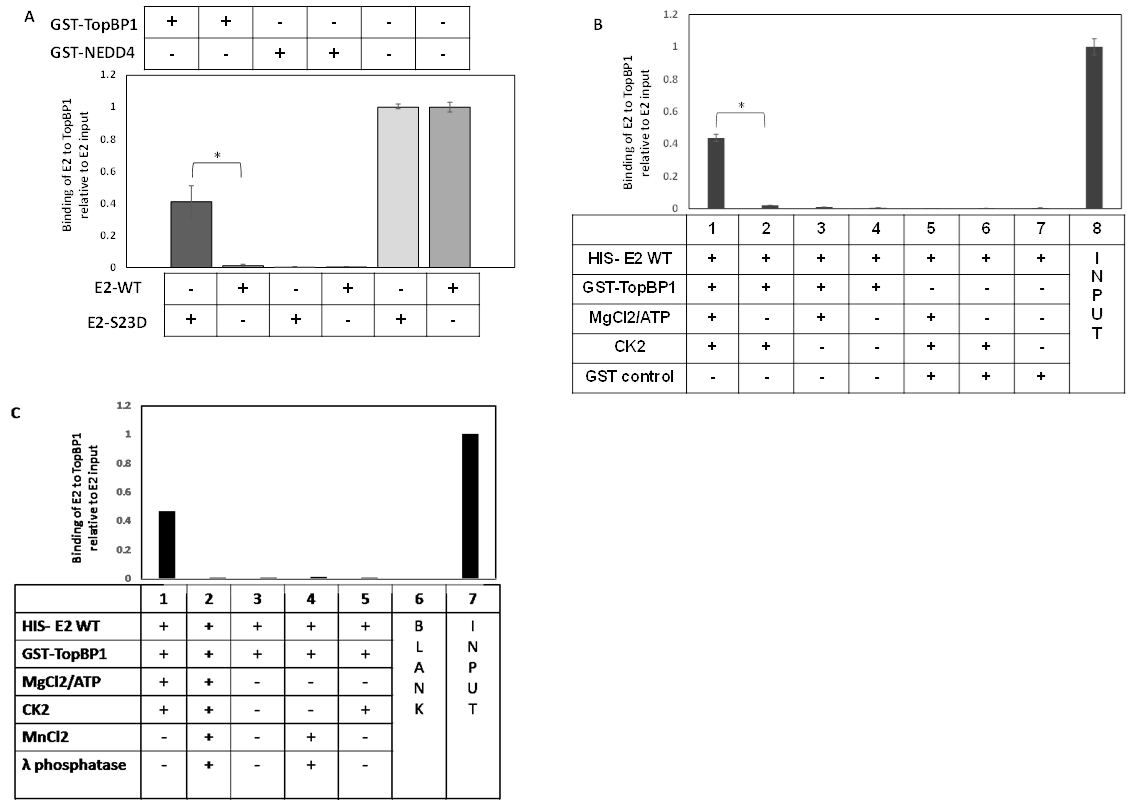

Supplement: FIG S1 [file mbio.01163-21-sf001.tif]

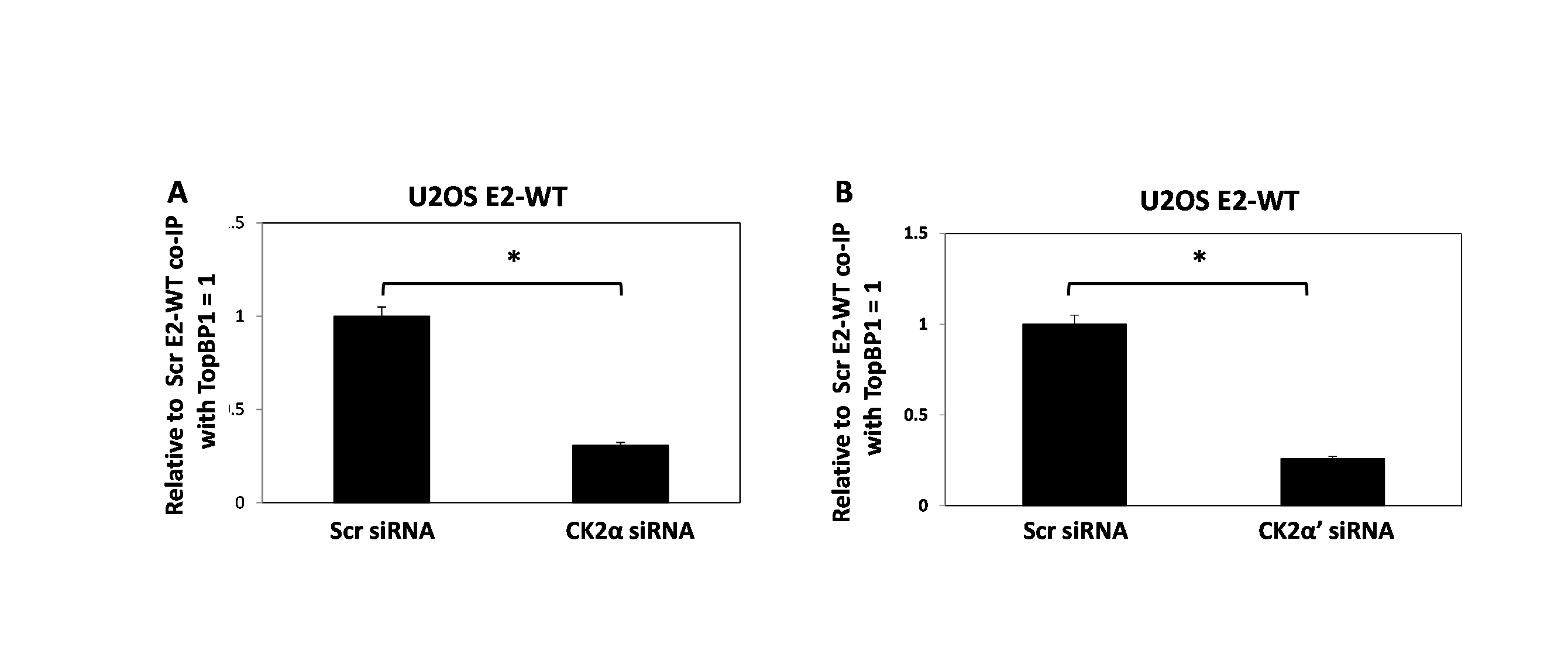

Supplement: FIG S2 [file mbio.01163-21-sf002.tif]

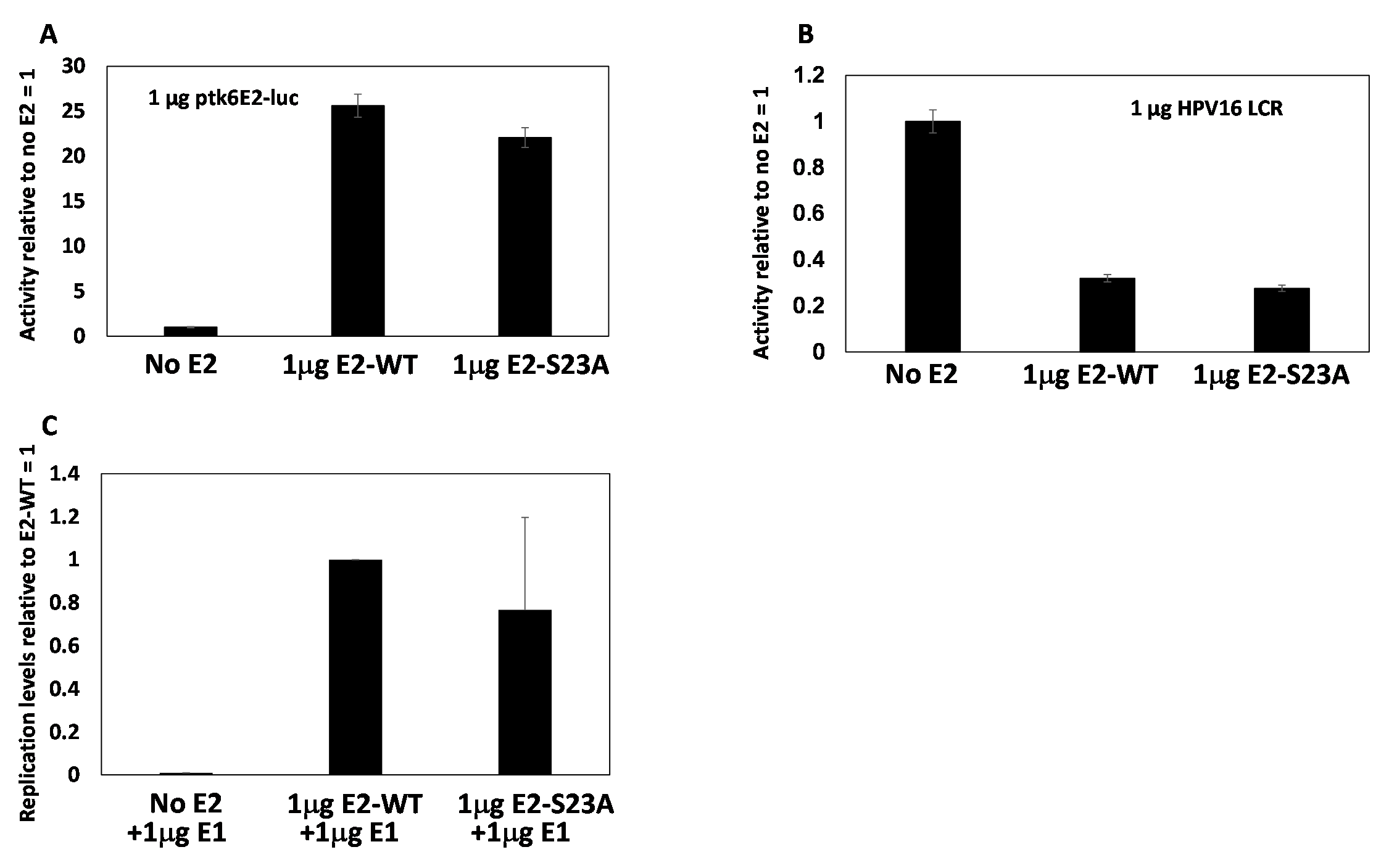

Supplement: FIG S3 [file mbio.01163-21-sf003.tif]

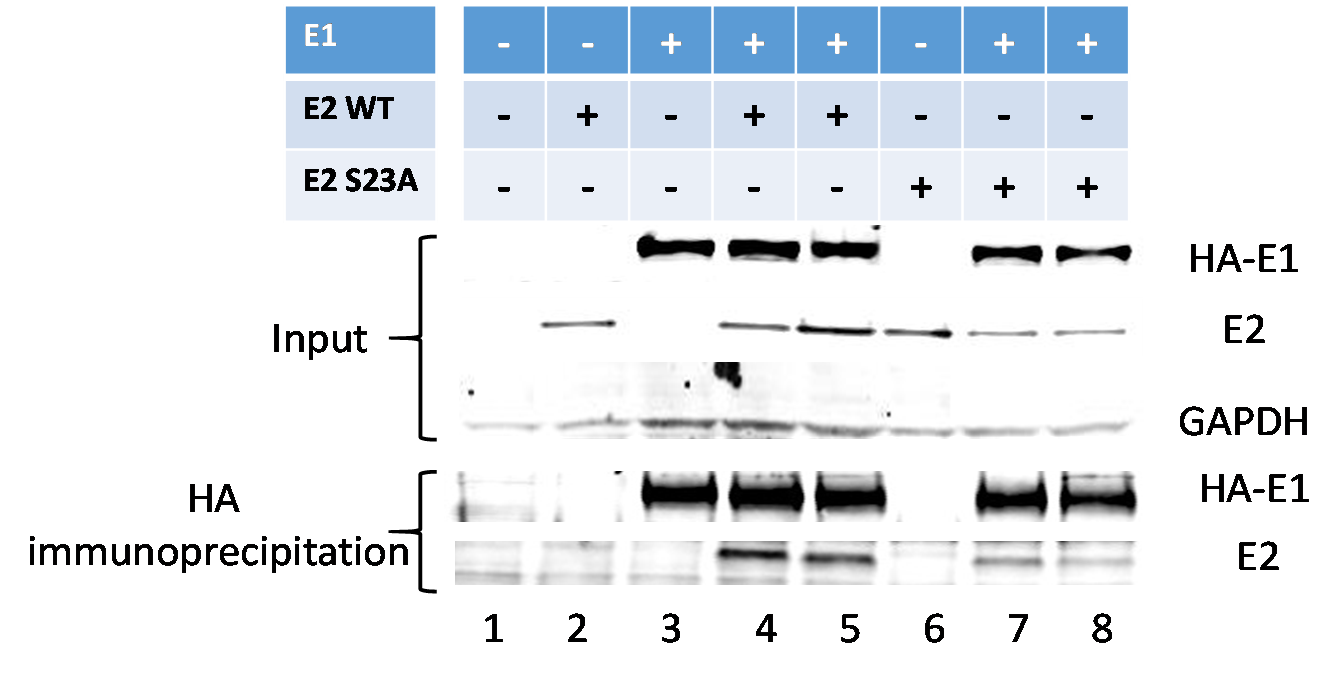

Supplement: FIG S4 [file mbio.01163-21-sf004.tif]

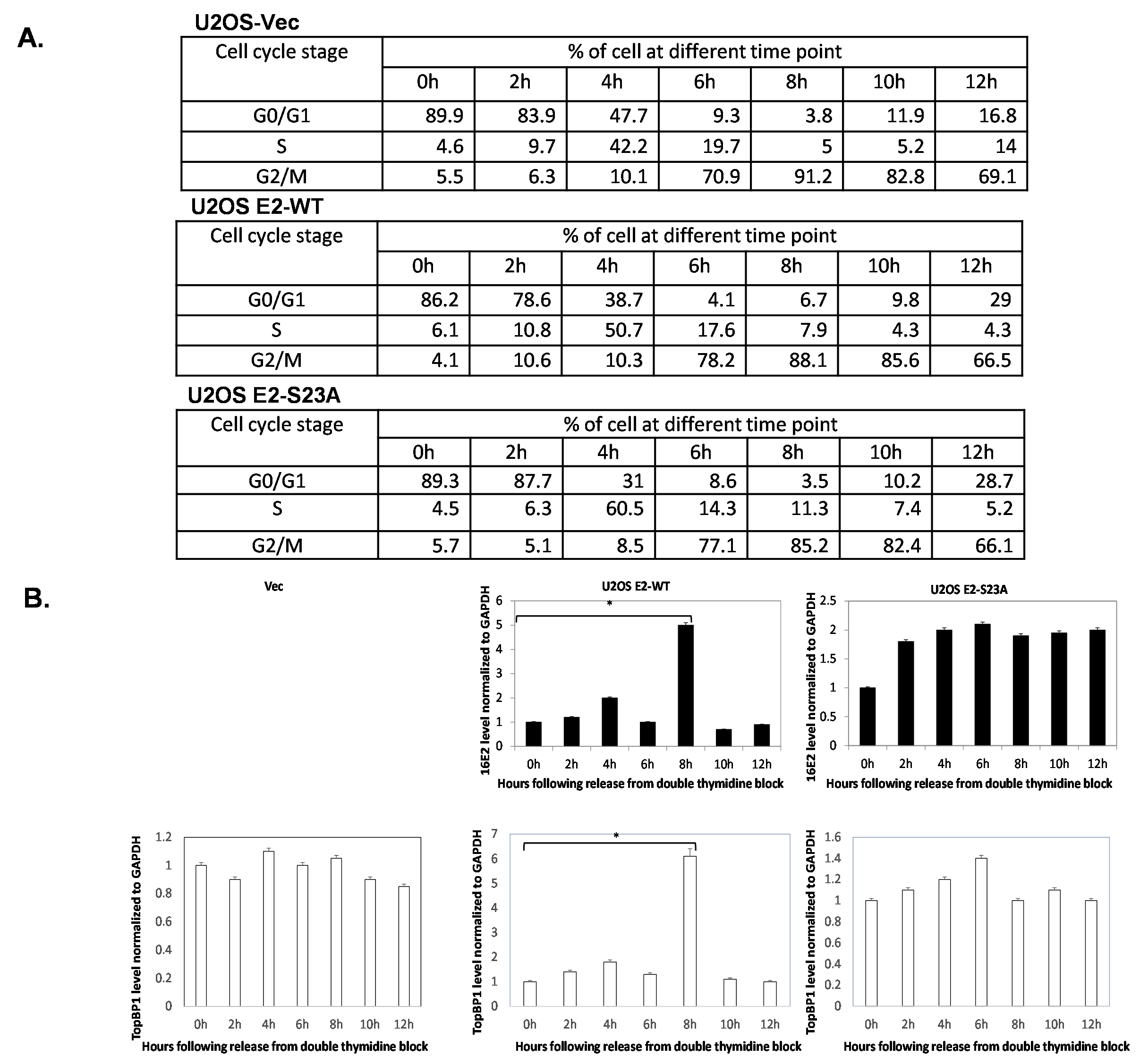

Supplement: FIG S5 [file mbio.01163-21-sf005.tif]

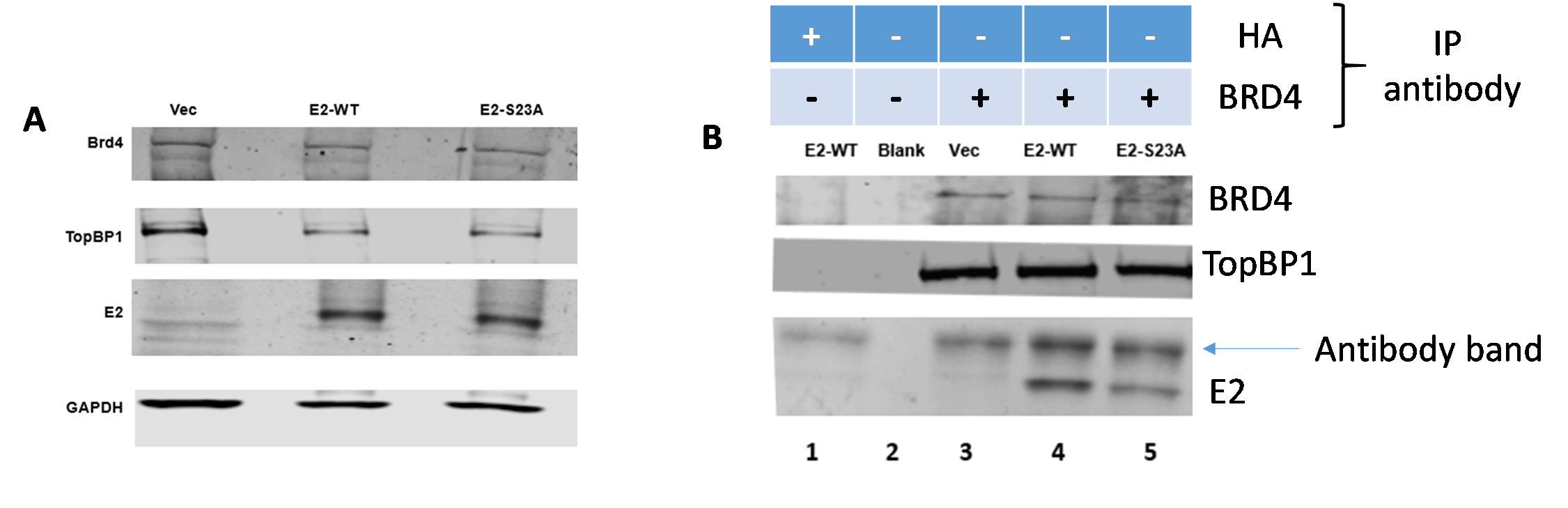

Supplement: FIG S6 [file mbio.01163-21-sf006.tif]

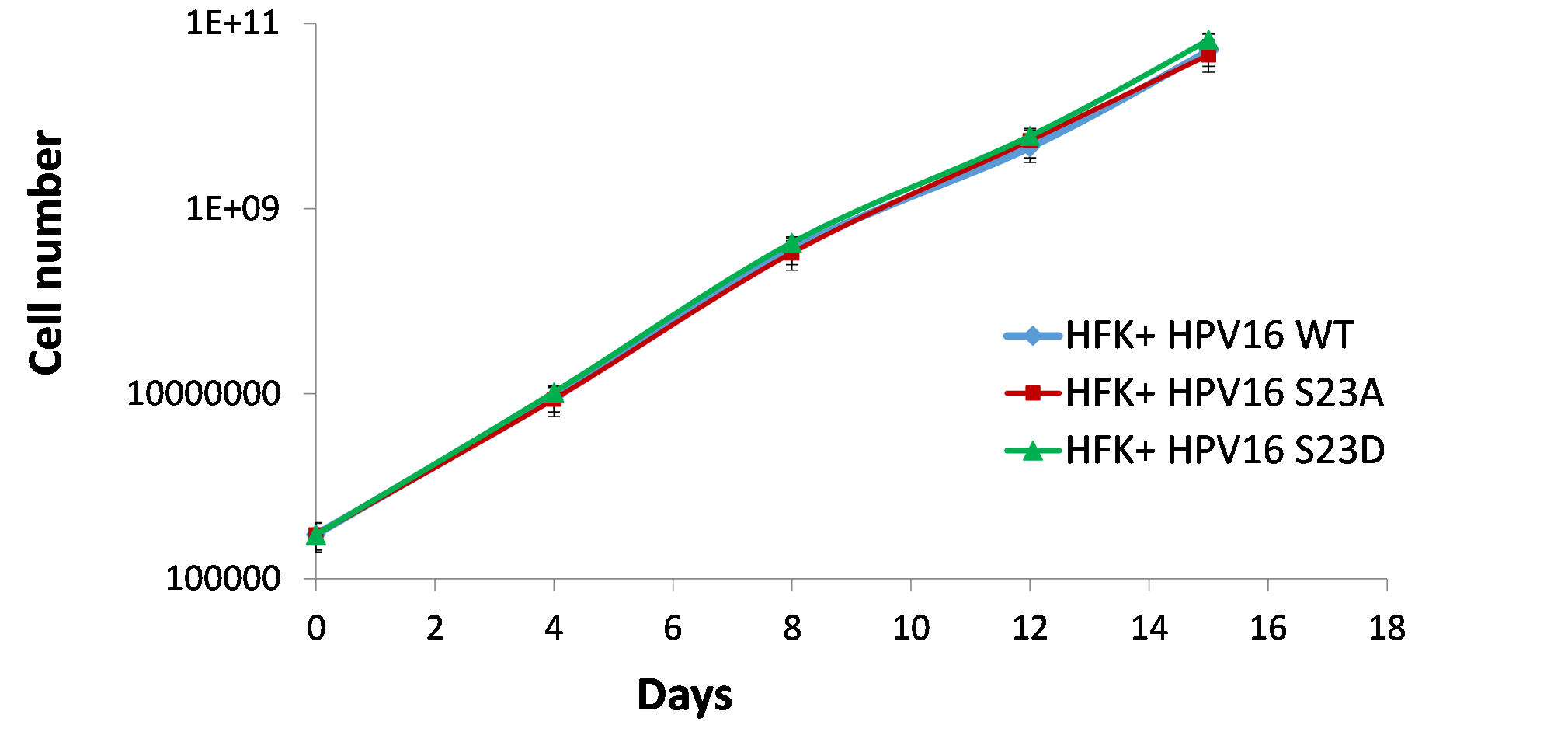

Supplement: FIG S7 [file mbio.01163-21-sf007.tif]

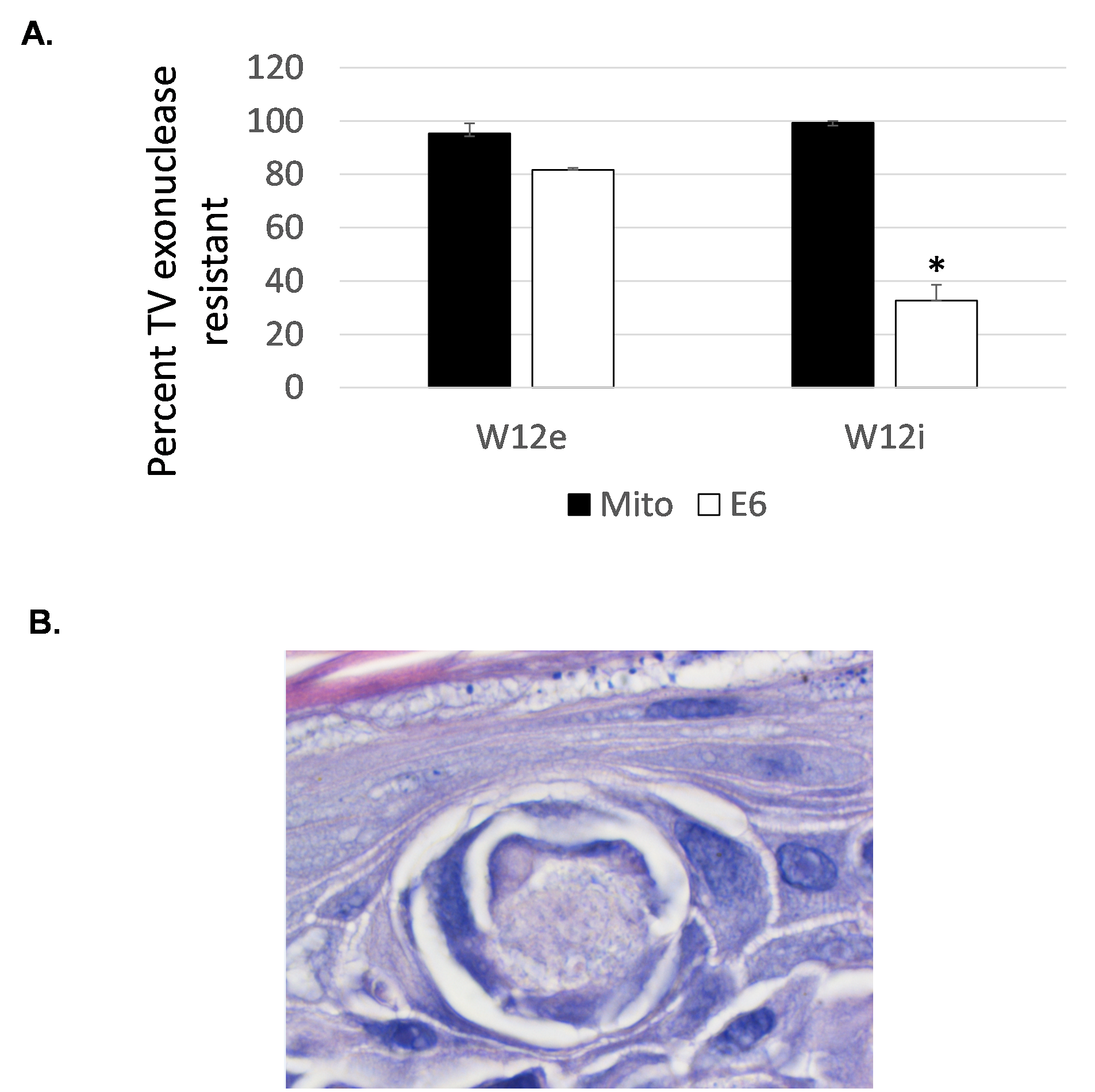

Supplement: FIG S8 [file mbio.01163-21-sf008.tif]

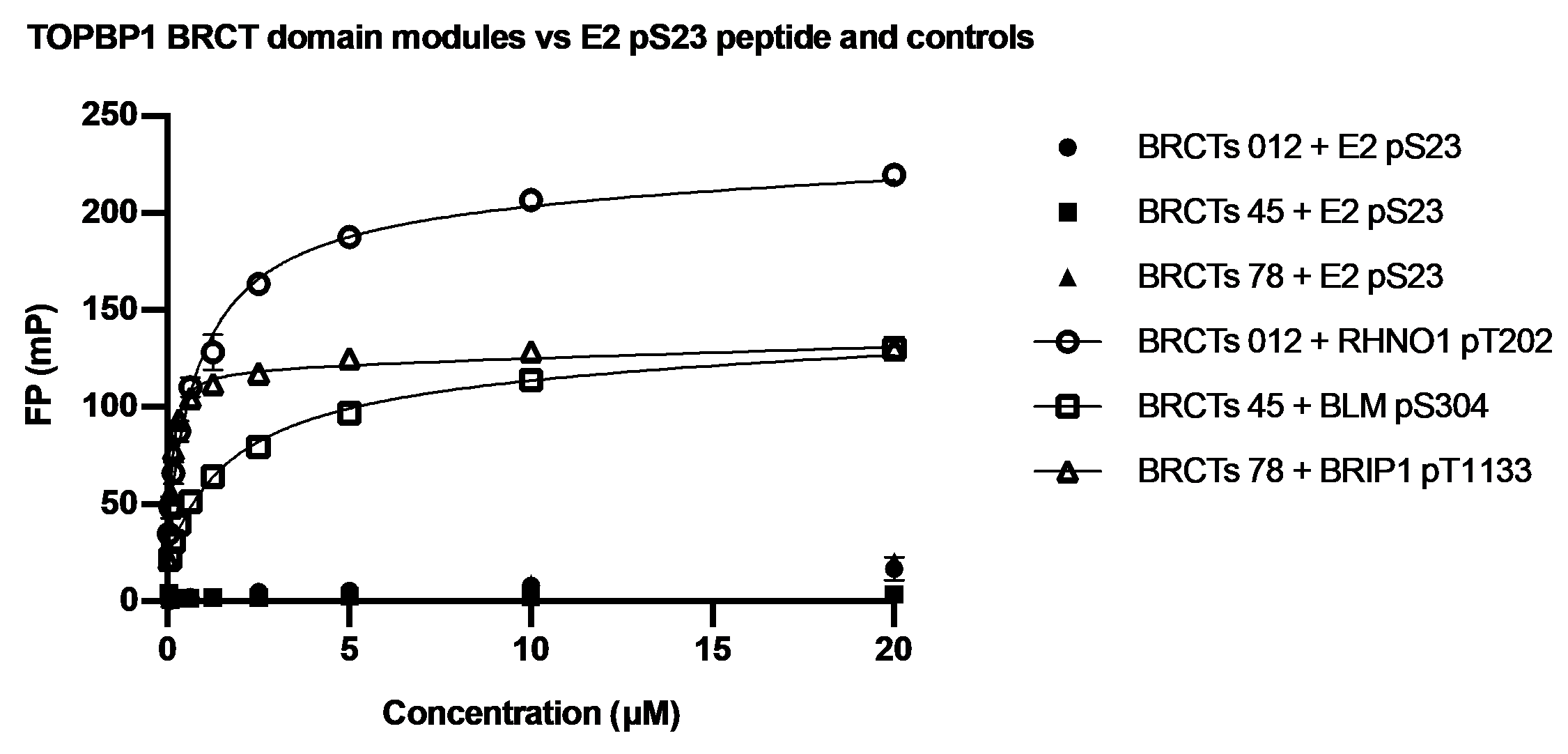

Supplement: FIG S9 [file mbio.01163-21-sf009.tif]
